# Temporal dynamics of cholinergic activity in the septo-hippocampal system

**DOI:** 10.3389/fncir.2022.957441

**Published:** 2022-08-25

**Authors:** Jeffrey D. Kopsick, Kyle Hartzell, Hallie Lazaro, Pranav Nambiar, Michael E. Hasselmo, Holger Dannenberg

**Affiliations:** ^1^Department of Bioengineering, George Mason University, Fairfax, VA, United States; ^2^Interdisciplinary Program for Neuroscience, George Mason University, Fairfax, VA, United States; ^3^Center for Systems Neuroscience, Boston University, Boston, MA, United States; ^4^Department of Psychological and Brain Sciences, Boston University, Boston, MA, United States

**Keywords:** medial septum, acetylcholine, running speed, darkness, temporal dynamics, fiber photometry, hippocampus, entorhinal cortex

## Abstract

Cholinergic projection neurons in the medial septum and diagonal band of Broca are the major source of cholinergic modulation of hippocampal circuit functions that support neural coding of location and running speed. Changes in cholinergic modulation are known to correlate with changes in brain states, cognitive functions, and behavior. However, whether cholinergic modulation can change fast enough to serve as a potential speed signal in hippocampal and parahippocampal cortices and whether the temporal dynamics in such a signal depend on the presence of visual cues remain unknown. In this study, we use a fiber-photometric approach to quantify the temporal dynamics of cholinergic activity in freely moving mice as a function of the animal’s movement speed and visual cues. We show that the population activity of cholinergic neurons in the medial septum and diagonal band of Broca changes fast enough to be aligned well with changes in the animal’s running speed and is strongly and linearly correlated to the logarithm of the animal’s running speed. Intriguingly, the cholinergic modulation remains strongly and linearly correlated to the speed of the animal’s neck movements during periods of stationary activity. Furthermore, we show that cholinergic modulation is unaltered during darkness. Lastly, we identify rearing, a stereotypic behavior where the mouse stands on its hindlimbs to scan the environment from an elevated perspective, is associated with higher cholinergic activity than expected from neck movements on the horizontal plane alone. Taken together, these data show that temporal dynamics in the cholinergic modulation of hippocampal circuits are fast enough to provide a potential running speed signal in real-time. Moreover, the data show that cholinergic modulation is primarily a function of the logarithm of the animal’s movement speed, both during locomotion and during stationary activity, with no significant interaction with visual inputs. These data advance our understanding of temporal dynamics in cholinergic modulation of hippocampal circuits and their functions in the context of neural coding of location and running speed.

## Introduction

Acetylcholine is an important neuromodulator of cognitive functions and behavior (Hasselmo, 2006; Picciotto et al., 2012) that are related to the processing of sensory information, memory, and navigation of physical and abstract mental spaces (Solari and Hangya, 2018). Conversely, cholinergic dysfunctions are a hallmark of many neurological and psychiatric diseases that affect memory and the coding of spatial information and sensory information, such as Alzheimer’s disease (Davies and Maloney, 1976; Hampel et al., 2018), autism spectrum disorders (Karvat and Kimchi, 2014), schizophrenia (Higley and Picciotto, 2014), and depression (Higley and Picciotto, 2014). Experimental data from animal models demonstrate a role of acetylcholine in modulating learning and memory (Hasselmo, 2006), attention to sensory stimuli (Hasselmo and McGaughy, 2004; Yu and Dayan, 2005; Pinto et al., 2013), visual cue detection (Gritton et al., 2016), and brain state transitions between waking, sleep, or behavioral states (Marrosu et al., 1995; Xu et al., 2015; Harrison et al., 2016). In the hippocampal formation, cholinergic modulation of network dynamics, synaptic plasticity, and neuronal excitability supports the formation of spatial memories and cognitive functions supporting memory-guided navigation (Blokland et al., 1992; Ohno et al., 1993, 1994; Stancampiano et al., 1999; Dannenberg et al., 2017). In particular, experimental data (Rogers and Kesner, 2003) and computational models (Hasselmo, 1999, 2006) propose an important role of acetylcholine in separating the encoding and retrieval of memory traces. The primary and major source of cholinergic innervation of the hippocampal formation is provided by cholinergic projection neurons in the medial septum/diagonal band of Broca (MSDB) (Mesulam et al., 1983; Rye et al., 1984). MSDB cholinergic projection neurons have a key function in modulating hippocampal activity *via* a direct and an indirect pathway (Alreja et al., 2000; Wu et al., 2000, 2003; Dannenberg et al., 2015). The indirect pathway is particularly important for modulating theta (6–10 Hz) rhythmic activity in the hippocampal local field potential *via* the modulation of glutamatergic and GABAergic projection neurons within the MSDB. Both the frequency and amplitude of theta rhythmic activity are correlated with the running speed of an animal (Whishaw and Vanderwolf, 1973), and manipulations that disrupt either the power of theta (Brandon et al., 2011; Koenig et al., 2011) or the relationship to running speed of theta rhythmic frequency (Winter et al., 2015; Dannenberg et al., 2020) disrupt the coding of location by grid cells in the medial entorhinal cortex. Models of memory-guided navigation therefore propose a role of cholinergic modulation in the coding of location and running speed in the hippocampal formation (Dannenberg et al., 2016). In fact, cholinergic activity has recently been demonstrated to be correlated with running speed in mice (Jing et al., 2020; Zhang et al., 2021). However, the temporal dynamics in cholinergic activity and how they relate to changes in running speed and behavioral activities remain elusive, despite an ongoing debate in the field about the role of slow vs. fast time scales in cholinergic modulation (Disney and Higley, 2020; Sarter and Lustig, 2020). To test the hypothesis that cholinergic activity changes fast enough to serve as a potential code for running speed in the hippocampal formation, we used a fiber-photometric approach (Gunaydin et al., 2014; Lerner et al., 2015) to monitor the population activity of cholinergic projection neurons in the MSDB of freely behaving mice. We next tested whether the activity of the septo-hippocampal cholinergic system is a function of characteristic behaviors, including stationary activities such as grooming and rearing. Lastly, we analyzed whether the observed temporal dynamics in cholinergic activity were a function of visual cues. The presented results demonstrate that the activity of the septo-hippocampal cholinergic system (i) changes fast enough to match changes in running speed, (ii) is strongly and linearly correlated to the logarithm of the animal’s running speed, (iii) remains strongly correlated to the animal’s neck movements during stationary activity, (iv) is elevated during rearing, and (v) remains unchanged during darkness.

## Materials and methods

### Animals

Before surgery, mice were habituated to the experimenter and testing room over at least 1 week by handling mice at least once daily inside the testing room. During handling, mice were gently removed repeatedly from their home cage and held in the experimenter’s hand. All experimental procedures were approved by the Institutional Animal Care and Use Committee for the Charles River Campus at Boston University. Mice were purchased from The Jackson Laboratory (Wildtype, C57Bl/6J; ChAT-IRES-Cre, B6;129S6-Chat^TM 2(cre)Lowl^/J). Transgenic mice were maintained as homozygous, and heterozygous mice of both sexes were used for experiments. For data collection, adult mice were housed in Plexiglas cages together with their siblings prior to surgery, but separated for individual housing after surgery, and maintained on a reversed 12-h light/12-h dark cycle. The housing cages contained a spherical treadmill that provided mice the opportunity to exercise.

### Viral transduction and optical fiber implantation

Mice were injected with buprenorphine (0.1-mg/kg, s.c.) and atropine (0.1-mg/kg, i.p.), and survival surgery was performed under isoflurane anesthesia for virus injection and optical fiber implantation targeting the MSDB. The carrier gas for isoflurane was 100% oxygen. Gas anesthesia was induced by placing the mice in an induction chamber and filling the chamber with 3% isoflurane. Anesthesia was maintained throughout the surgery at an isoflurane concentration between 1.0 and 1.5% delivered *via* a nose cone. Body temperature was maintained and monitored throughout the surgery *via* a heating pad and a homeothermic monitoring system (Harvard Apparatus, Holliston, MA). Four anchoring screws were positioned across the skull. For cell-type specific targeting of cholinergic MSDB neurons, we used stereotactically targeted virus injections of rAAV S1 FLEX-CAG-jGCaMP7s-WPRE (Lot v28549, Addgene, #104495 AAV-1) into the MSDB of 3–6 months old ChAT-Cre mice. 2 × 250-nl virus solution was injected at two ventral sites within the MSDB. To that end, a craniotomy was performed 1.2-mm anterior and 0.7-mm lateral to Bregma, and the injection needle was lowered 4.8 and 4.4-mm at a 10° polar and –90° azimuth angle, following stereotactic coordinates from Paxinos and Franklin (2008). The injection needle (34-g, beveled, WPI) was left in place for 3 and 5 min after the first and second injections (100-nl/min, UMP3 electrical pump, WPI) to prevent backflow of the injected virus solution. After virus injection was complete, the same opening in the skull was used to implant an implantable optical fiber (total diameter: 230-μm; 200-μm core, N.A. 0.48; MFC_200/230-0.48_8mm_SMR_FLT; Doric lenses), 1.2-mm anterior and 0.7-mm lateral to Bregma. The optical fiber was lowered 4.2-mm from the brain surface at an 10° polar and -90° azimuth angle and cemented on the animal’s skull with dental acrylic that was blackened by mixing in graphite. Animals were given buprenorphine (0.1-mg/kg, i.p.), enrofloxacin (7.5-mg/kg, i.p.), and ketoprofen (3-mg/kg, i.p.) during a 5-day postsurgical care period and allowed 1 week in total to fully recover after surgery before beginning of recordings.

### Behavioral testing

All recordings were performed while animals foraged for small pieces of Froot Loops (Kellog Company, Battle Creek, MI, United States) in the open-field environment. The fiber photometry experiments were performed within a time window of ∼1–3 weeks after the virus injection. During that time window, levels of jGCaMP7s expression where high enough to result in a good signal-to-noise ratio but low enough for cell toxicity to occur. A typical recording session lasted between 5 and 15-min. A rectangular housing cage (34-cm × 28-cm) with transparent 20-cm high walls (*n* = 1 mouse) or an acrylic box (40-cm × 40-cm) with black 30-cm high walls and a visual cue card (*n* = 4 mice) were used as open field environments. The recording room contained no windows and was shielded with a laser-proof black curtain. Recording sessions of the one mouse foraging in the rectangular housing cage were performed during standard lighting conditions only. Recordings of the four other mice foraging in the acrylic box were performed during standard lighting conditions and darkness. On a typical day, four recording sessions were performed. The first and last sessions were performed during standard room lighting with the room ceiling light turned on, and the second and third sessions were recorded during darkness with the room ceiling light turned off. The experimental arena was cleaned with 70% isopropanol followed by water between sessions and mice. Animals of both sexes were included in this study. The phase of estrous cycle was not checked in female mice before experiments.

### Histology

After data acquisition was finished, animals were deeply anesthetized by intraperitoneal injection of Euthasol (390 mg/kg) and transcardial perfusion was performed with Dulbecco’s phosphate buffered saline (DPBS) containing 0.9% calcium chloride followed by 10% buffered formalin (SF100-4, Thermo Fisher Scientific). Brains were extracted and stored in fixative for 1 day. The MSDB was cut into 35-μm coronal sections using a vibratome. During the slicing, the tissue was submerged in DPBS containing 0.9% calcium chloride. For immunolabeling of ChAT in MSDB neurons, slices were incubated for 2 days at 4 degrees Celsius in wells of a 48-well plate with goat anti-ChAT affinity purified polyclonal antibody (catalog #AB 144P, Merck Millipore, diluted 1:200) and 0.3% Triton X-100 (Sigma-Aldrich, St. Louis, MO), washed three times for 15-min with DPBS containing 0.9% calcium chloride at room temperature, and incubated for 2 h at room temperature with a secondary antibody (Cy3-conjugated donkey anti-goat IgG polyclonal antibody, catalog #AP180C, Merck Millipore, diluted 1:400). Slices were washed three times again and mounted in Aqua-Poly/Mount (#18606-20, Polysciences, Inc., Warrington, PA).

### Fiber photometry system

We used a custom-built fiber photometry system inspired by Gunaydin et al. (2014) and Lerner et al. (2015). The fiber photometry system used a 473-nm Omnicron Luxx laser modulated at 211-Hz and a 405-nm UV LED (Thorlabs, M405FP1) modulated at 531-Hz using optical choppers. Excitation at 405-nm served as an isosbestic control signal. Laser light was delivered into the brain *via* a system of fiber patch cords (Thorlabs) and a rotary joint (FRJ_1x1_FC-FC, Doric lenses). The laser light power entering the implanted optical fiber was measured before and after every recording session and adjusted before the recording session to yield an estimate of 40–60 μW laser power delivered into the MSDB. Data were acquired at a sampling rate of 500-Hz.

### Video tracking

Mice were video-tracked using a thermal camera (FLIR, SC8000) at a video frame rate of 26–30 frames/s controlled *via* TTL pulses generated by an external signal generator. Temperature values were color-coded with a gray scale and exported in mpeg-4 video format. TTL pulses sent from the camera were recorded along with the fiber-photometry system to synchronize the videos with the fiber-photometry signal. For three mice, care was taken that camera settings remained constant (same position over the open field environment, same angle of view, same zoom lens setting, and same sampling rate of 30 frames/s). Data from these three mice were used for VAME analysis.

### Analysis of fiber photometry data

Data were re-sampled to match the 30-Hz video frame rate used for video-tracking of animal pose and behavior. The fiber-photometry signal was then processed to adjust for (i) photobleaching that results in an exponential decay of the signal, and (ii) motion artifacts employing the use of an isosbestic control signal. This was done in the following way. First, we subtracted the isosbestic control signal from the jGCaMP7s signal and fitted a 2nd-degree polynomial curve to that result. Next, we added the fitted curve to the isosbestic control signal, resulting in an adjusted control signal (see Supplementary materials for examples of raw traces). We then formulated an optimization problem to find the two parameters a and b that minimized the term ∑(*s* − (c*α + β))^2^, where _s_ stands for the jGCaMP7s signal, and _c_ stands for the adjusted control signal. As a final step, we computed DF/F as $\frac{s-\left(c^{*}\alpha +\beta \right)}{c^{*}\alpha +\beta }$ . For analyses that average or compare data across sessions, the z-scored ΔF/F was used to account for variations in signal strength across sessions. The z-scored ΔF/F was computed as $\frac{\left(x-\bar{x}\right)}{s_{\text{x}}}$, where x stands for ΔF/F, $\bar{x}$ for the mean, and s_*x*_ for the standard deviation of ΔF/F over time.

### Markerless pose estimation and calculation of running speed

We used the deep learning tool DeepLabCut (Mathis et al., 2018) for markerless pose estimation of the animal’s neck, nose, left ear, right ear, tail base, and tail tip. The neck position was used to estimate the animal’s movement speed. For later identification of behavioral motifs using variational animal motion embedding (VAME), we labeled the animal’s neck, nose, left ear, right ear, three points along the spine, tail base, two points along the tail, and the tail tip.

### Granger causality analysis

Granger causality analysis was performed using the MATLAB MVGC multivariate Granger causality toolbox developed by Barnett and Seth (2014). Granger causality analysis was performed on the first 5-min of each recording session to enable statistical comparison across sessions and mice.

### Variational animal motion embedding

To identify behavioral motifs and larger clusters of behavioral motifs (“communities”), we used the deep learning tool VAME (Luxem et al., 2022). All sessions that were included in this analysis were performed with the exact same camera setup and settings. Supplementary Videos for each behavioral community are provided in Supplementary material related to Figures 4C–F. To create these videos, VAME produced a time series corresponding to a behavioral community label for each frame in the video sessions provided as input. This time series was then used to create behavioral community videos 40 s in length: the first entry where the behavioral community label appeared in the time series was used as the video’s first frame, with successive frames added until 40 s of the behavior were created—these videos thus constitute 40 s of behavioral community sequences appearing across video sessions.

**FIGURE 1:**
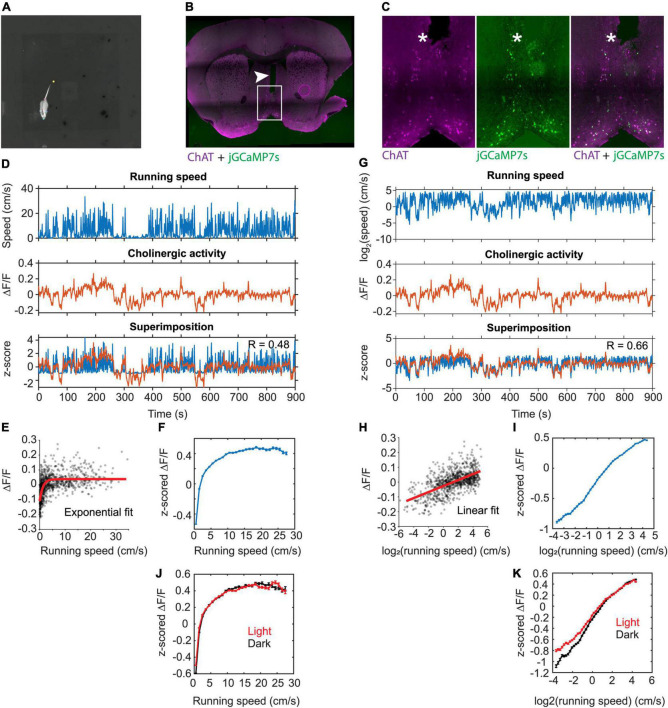
Linear correlation between the population activity of MSDB cholinergic neurons and the logarithm of an animal’s running speed. **(A)** One video frame showing the DeepLabCut labels for neck, nose, left ear, right ear, tail base, and tail tip. Gray scale of the video shows the temperature of the mouse recorded *via* a thermal camera. **(B)** Histological verification of the fiber track position in the MSDB. The arrowhead marks the fiber track. Magenta colors indicate immune-positive staining for ChAT, green colors indicate presence of jGCaMP7s. **(C)** Immuno-histological data on jGCaMP7s expression in the MSDB neurons. Note the overlap between jGCaMP7s-postive and ChAT-positive neurons. The asterisk marks the tip of the implanted optical fiber. **(D)** Data on running speed and activity of cholinergic neurons in the MSDB measured *via* fiber-photometry for one example session. Note the high fluctuations in the fiber photometry signal during low running speeds. **(E)** Scatter plot of cholinergic activity vs. running speed; data points were sampled in 1-s intervals from the time series data shown in (D). Red line shows the exponential fit to the data. **(F)** Speed tuning curve of cholinergic activity. Data show mean ± s.e.m. of speed-binned data with a bin width of 1-cm/s; data from 103 sessions recorded from 5 mice. **(G)** Same data as in (D) but now plotting the logarithm of running speed. **(H)** Scatter plot of cholinergic activity vs. the logarithm of the animal’s running speed; data points were sampled in 1-s intervals from the time series data shown in (G). Red line shows the linear fit to the data. **(I)** Tuning curve shows cholinergic activity as a function of the logarithm of running speed; data show mean ± s.e.m. of binned data with a bin width of 0.1; data from 103 sessions recorded from 5 mice; **(J)** Speed tuning curves of cholinergic activity comparing data from light (red, *n* = 55) and darkness (black, *n* = 47) sessions; data presented as in (F) **(K)** data presented as in (I) comparing data from light (red, *n* = 55) and darkness (black, *n* = 47) sessions; no significant difference between light and darkness sessions was found (see Table 2 for statistics). R, Pearson’s correlation coefficient.

**TABLE 1:** Number of sessions and time span of recordings per mouse.

Mouse ID	Sex	Number of sessions	Time span of recordings (days)
#611915	Female	20	12
#611916	Female	46	16
#611926	Male	8	10
#616195	Male	17	13
#616211	Male	12	15

Total number of sessions was 103 from 5 mice recorded over a time span of 10–15 days per mouse.

**TABLE 2:** Results of a linear mixed-effects model of the z-scored ΔF/F of cholinergic activity with three fixed effects, namely the y-intercept, the logarithm of allocentric neck movement speed, and the interaction between the logarithm of allocentric neck movement speed and darkness.

Fixed effects coefficients						
**Name**	**Estimate**	**SE**	**t-statistic**	**DF**	***P*-value**	**95% CI**
y-intercept	–0.169	0.072	–2.35	1.31 × 10^6^	0.0186	[–0.310 –0.028]
log_2_(speed)	0.221	0.019	11.92	1.31 × 10^6^	<0.0001	[0.185 0.258]
log_2_(speed)* Darkness	0.013	0.013	0.99	1.31 × 10^6^	0.3213	[–0.013 –0.038]

**Model formula**						
${}_{y_{imkl}=\beta_{0}+\beta_{1}log_{2}\left(speed\right)+\sum_{m=2}^{2}\beta_{2m}log_{2}{\left(speed\right)}_{\text{i}}I{\left[D\right]}_{\text{im}}+b_{0k}A_{\text{k}}+b_{0l}S_{\text{l}}+b_{1k}{\left(log_{2}\left(speed\right)\right)}_{\text{ik}}+b_{1l}{\left(log_{2}\left(speed\right)\right)}_{\text{il}}+b_{2k}{\left(log_{2}\left(speed\right)*I\left[D\right]\right)}_{\text{imk}}+b_{2l}{\left(log_{2}\left(speed\right)*I\left[D\right]\right)}_{\text{iml}}+\varepsilon_{\text{imkl}},}$ where index *i* corresponds to the # of observations, index *m* corresponds to light or darkness, index *k* corresponds to the animal identity, and index *l* corresponds to the session identity. *A*_*k*_ represents the *k*th animal, *S*_l_ represents the *l*th session, *I*[*D*]_im_ is the dummy variable representing level *m* of the darkness type, *I*[*C*]_*ik*_ the dummy variable representing level *k* of, *b* the random mixed effects, ε_*imkl*_ the observation error, and *y*_imkl_ the response variable representing z-scored ΔF/F. Random effects and observation error are normally distributed around zero.						
**Model statistics**						
AIC: 3.424 × 10^6^	BIC: 3.424 × 10^6^	Log-likelihood: -1.7 × 10^6^	Deviance: 3.424 × 10^6^			

Random effects of animal (n = 5) and session (n = 102, 55 “light” sessions and 47 “dark” sessions) on all fixed effects were included in addition to a random error term. Total number of observations: 1310717; Fixed effects coefficients: 3; Random effects coefficients: 321; Covariance parameters: 7. SE, standard error; DF, degrees of freedom; CI, confidence interval.

**FIGURE 2:**
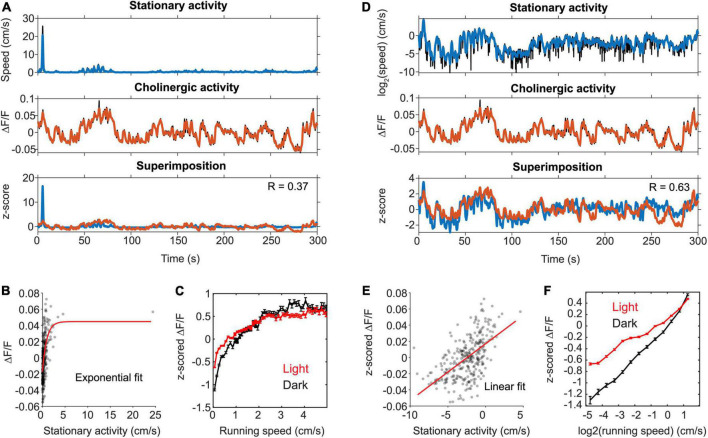
The activity of cholinergic neurons is linearly correlated to neck movements during stationary behavior. **(A)** Data on the speed of neck movements during stationary behaviors and activity of cholinergic neurons in the MSDB measured *via* fiber-photometry for one example session, in which the mouse remained stationary (running speed < 3-cm/s) for more than two thirds of the session length. Note the high fluctuations in the fiber photometry signal during stationary activity. **(B)** Scatter plot of cholinergic activity vs. running speed; data points were sampled in 1-s intervals from the time series data shown in (A). Red line shows the exponential fit to the data. **(C)** Speed tuning curves of cholinergic activity comparing data from light (red, data from 13 sessions, 3 mice) and darkness (black, data from 3 sessions, 2 mice) sessions. Data show mean ± s.e.m. of speed-binned data with a bin width of 1-cm/s. **(D)** Same data as in (A) but now plotting the logarithm of neck movement speed. **(E)** Scatter plot of cholinergic activity vs. the logarithm of the animal’s neck movement speed; data points were sampled in 1-s intervals from the time series data shown in (D). Red line shows the linear fit to the data. **(F)** Tuning curve shows cholinergic activity as a function of the logarithm of neck movement speed; data show mean ± s.e.m. of binned data with a bin width of 0.1; data on light sessions (red) from 13 sessions, 3 mice; data on darkness sessions (black) from 3 sessions, 2 mice; no significant difference between light and darkness sessions was found (see Table 3 for statistics). R, Pearson’s correlation coefficient.

**TABLE 3:** Results of a linear mixed-effects model of the z-scored ΔF/F of cholinergic activity during stationary behavioral activity with three fixed effects, namely the y-intercept, the logarithm of allocentric neck movement speed, and the interaction between the logarithm of allocentric neck movement speed and darkness.

Fixed effects coefficients						
**Name**	**Estimate**	**SE**	**t-statistic**	**DF**	***P*-value**	**95% CI**
y-intercept	0.232	0.127	1.83	1.46 × 10^5^	0.067	[–0.016 0.481]
log_2_(speed)	0.226	0.028	8.14	1.46 × 10^5^	<0.0001	[0.171 0.280]
log_2_(speed)* Darkness	0.068	0.050	1.37	1.46 × 10^5^	0.170	[–0.029 0.166]

**Model formula**						
${\text{y}}_{\text{imkl}}{\text{= β}}_{\text{0}}{\text{+β}}_{\text{1}}{\text{log}}_{\text{2}}\text{(speed)+}\overset{2}{\underset{\text{m=2}}{\sum }}{\text{β}}_{\text{2m}}{\text{log}}_{\text{2}}{\left(\text{speed}\right)}_{\text{i}}{\text{I[D]}}_{\text{im}}{\text{+b}}_{\text{0k}}{\text{A}}_{\text{k}}{\text{+b}}_{\text{0l}}{\text{S}}_{\text{l}}{\text{+ b}}_{\text{1k}}{{\text{(log}}_{\text{2}}\left(\text{speed}\right)\text{)}}_{\text{ik}}{\text{+b}}_{\text{1l}}{{\text{(log}}_{\text{2}}\left(\text{speed}\right)\text{)}}_{\text{il}}{\text{+b}}_{\text{2k}}{{\text{(log}}_{\text{2}}\left(\text{speed}\right)*\text{I[D]})}_{\text{imk}}{\text{+b}}_{\text{2l}}{{\text{(log}}_{\text{2}}\left(\text{speed}\right)*\text{I[D])}}_{\text{iml}}{\text{+ ε}}_{\text{imkl}}\text{,}$ where index *i* corresponds to the # of observations, index *m* corresponds to light or darkness, index *k* corresponds to the animal identity, and index *l* corresponds to the session identity. *A*_*k*_ represents the *k*th animal, *S*_*l*_ represents the *l*th session, *I*[*D*]_*im*_ is the dummy variable representing level *m* of the darkness type, *I*[*C*]_*ik*_ the dummy variable representing level *k* of, *b* the random mixed effects, ε_*imkl*_ the observation error, and *y*_*imkl*_ the response variable representing z-scored ΔF/F. Random effects and observation error are normally distributed around zero.						
**Model statistics**						
AIC: 3.597 × 10^5^	BIC: 3.598 × 10^5^	Log-likelihood: -1.8 × 10^5^	Deviance: 3.597 × 10^5^			

Data included in this model are from a subset of n = 16 out of 103 sessions in which the mouse was stationary (allocentric neck movement speed < 3-cm/s) for more than two thirds of the total length of the recording session. Random effects of the animal (n = 3) and sessions (n = 16, 13 “light” sessions and 3 “dark” sessions) on all fixed effects were included in addition to a random error term. Total number of observations: 146,234; Fixed effects coefficients: 3; Random effects coefficients: 57; Covariance parameters: 7. SE, standard error; DF, degree of freedom; CI, confidence interval.

**FIGURE 3:**
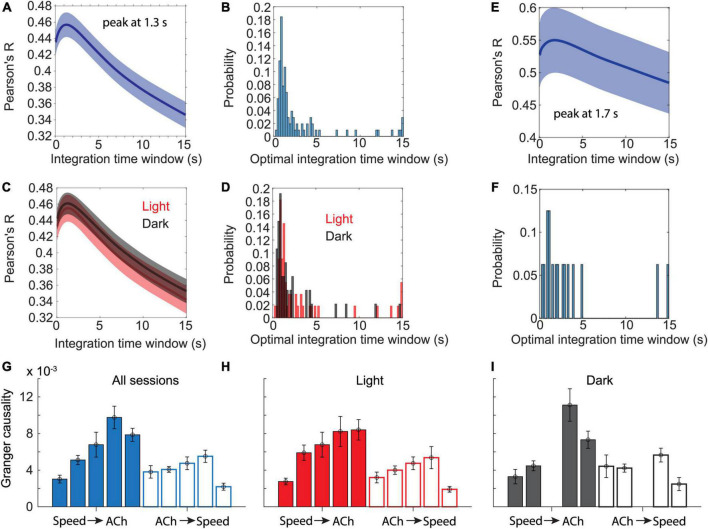
Temporal dynamics in cholinergic activity align with changes in movement speed. **(A)** Data show Pearson’s correlation coefficients between moving averages of cholinergic activity and the animal’s movement speed as a function of the time window used for computing the moving average of cholinergic activity. Blue line and shaded area show the mean ± s.e.m. of data from *n* = 103 sessions from five mice. The average peaks at 1.3-s. **(B)** Distribution of the optimal time windows, computed for each session, that maximize the correlation between moving averages of cholinergic activity and movement speed. The histogram shows a peak between 0.8 and 1-s. **(C,D)** Data presented as in (A,B) but comparing data from light (red, *n* = 55 sessions, five mice) and darkness sessions (black, *n* = 47, 4 mice). No significant difference was found between light and darkness sessions shown in (C) when comparing values at 1.3-s, *t*(100) = -0.192, *p* = 0.85. **(E)** Subset of data presented in (A) that only includes n = 16 sessions from three mice, in which the mice spent at least two thirds of the session time engaged in stationary behaviors (running speed < 3-cm/s). The average peaks at 1.7-s. **(F)** Same subset of data on stationary activity as shown in (E). Histogram shows the distribution of the optimal time windows, computed for each session, that maximize the correlation between moving averages of cholinergic activity and neck movement speed during stationary activity. **(G-I)** Granger causality magnitudes comparing Granger causality directions from the logarithm of movement speed to cholinergic activity (Speed → ACh) and vice versa (ACh → Speed) across animals. Each bar shows data from one mouse. Data are represented as mean ± s.e.m. across sessions. **(G)** Data from all sessions. **(H)** Data on light sessions only. **(I)** Data on dark sessions only. Note that one mouse was not recorded during darkness (see Tables 4, 5 and Supplementary Table 1 for statistics).

**TABLE 4:** Results of Granger causality analysis between the logarithm of movement speed and the fiber photometry signal (as z-scored ΔF/F) of cholinergic activity in the MSDB.

Mouse	GC, Speed→ACh	GC, ACh→Speed	Model order	*P*-value, Speed→ACh	*P*-value, ACh→Speed
#611915	0.00153	0.00149	9	<0.0001	<0.0001
#611916	0.00275	0.00141	9	<0.0001	<0.0001
#611926	0.00363	0.00239	9	<0.0001	<0.0001
#616195	0.00603	0.00194	20	<0.0001	<0.0001
#616211	0.00268	0.00015	9	<0.0001	0.0649

GC, Granger causality magnitude. Speed → ACh represents the direction from the logarithm of movement speed to cholinergic activity; conversely, ACh → Speed represents the direction from cholinergic activity to the logarithm of movement speed. This analysis treated the sessions from an individual mouse as multi-trial data from that mouse. See Table 1 for information on the number of sessions per individual mouse.

**TABLE 5:** Results of a linear mixed-effects model of Granger causality between the logarithm of movement speed (Speed) and the z-scored ΔF/F of cholinergic activity (ACh) with four fixed effects, namely the y-intercept, the Granger causality magnitude with two levels of direction, namely from the logarithm of movement speed to cholinergic activity (Speed → ACh) and from cholinergic activity to the logarithm of movement speed (ACh → Speed), lighting condition with two levels, namely light and darkness, and the interaction between direction in Granger causality and lighting condition.

Fixed effects coefficients						
**Name**	**Estimate**	**SE**	**t-statistic**	**DF**	***P*-value**	**95% CI**
y-intercept	0.00635	0.00105	6.05	200	<0.0001	[0.00428 0.00842]
ACh –> Speed	–0.00198	0.00105	–1.88	200	0.061	[–0.00406 9.48 × 10^–05^]
Darkness	0.00018	0.00061	0.29	200	0.773	[–0.00102 0.00137]
(ACh –> Speed)*Darkness	–0.00065	0.00068	–0.96	200	0.338	[0.33829 –0.00199]

**Model formula**						
${\text{y}}_{\text{ijmkl}}{\text{= β}}_{\text{0}}\text{+}\overset{2}{\underset{\text{j=2}}{\sum }}{\text{β}}_{\text{1j}}{\text{I[S]}}_{\text{ij}}\text{+}\overset{2}{\underset{\text{m=2}}{\sum }}{\text{β}}_{\text{2m}}\text{I}{\left[\text{D}\right]}_{\text{im}}\text{+}\overset{2}{\underset{\text{j=2}}{\sum }}\overset{2}{\underset{\text{m=2}}{\sum }}{\text{β}}_{\text{3jm}}\text{I}{\left[\text{S}*\text{D}\right]}_{\text{ijm}}{\text{+b}}_{\text{0k}}{\text{A}}_{\text{k}}{\text{+b}}_{\text{0l}}{\text{S}}_{\text{l}}{\text{+ b}}_{\text{1kj}}{\text{S}}_{\text{kj}}{\text{+b}}_{\text{1lj}}{\text{S}}_{\text{lj}}{\text{+b}}_{\text{2km}}{\text{D}}_{\text{km}}{\text{+b}}_{\text{2lm}}{\text{D}}_{\text{lm}}{\text{+b}}_{\text{3kjm}}{\text{(S}*\text{D)}}_{\text{kjm}}{\text{+b}}_{\text{3ljm}}{\text{(S}*\text{D})}_{\text{ljm}}{\text{+ ε}}_{\text{ijmkl}}\text{,}$ where index *i* corresponds to the # of observations, index *j* corresponds to the direction of Granger causality, index *m* corresponds to light or darkness, index *k* corresponds to the animal identity, and index *l* corresponds to the session identity. *A*_*k*_ represents the *k*th animal, *S*_*l*_ represents the *l*th session, *I*[*S*]_*ij*_ is the dummy variable representing level j of direction in Granger causality, *I*[*D*]_*im*_ is the dummy variable representing level *m* of the darkness type, *b* the random mixed effects, ε_*ijmkl*_ the observation error, and *y*_*ijmkl*_ the response variable representing z-scored ΔF/F. Random effects and observation error are normally distributed around zero.						
**Model statistics**						
AIC: -1762.5	BIC: -1719.4	Log-likelihood: 894.26	Deviance: -1788.5			

Random effects of the animal (n = 5) and sessions (n = 102, 55 “light” sessions and 47 “dark” sessions) on all fixed effects were included in addition to a random error term. Total number of observations: 204; Fixed effects coefficients: 4; Random effects coefficients: 428; Covariance parameters: 9. SE, standard error; DF, degree of freedom; CI, confidence interval.

**FIGURE 4:**
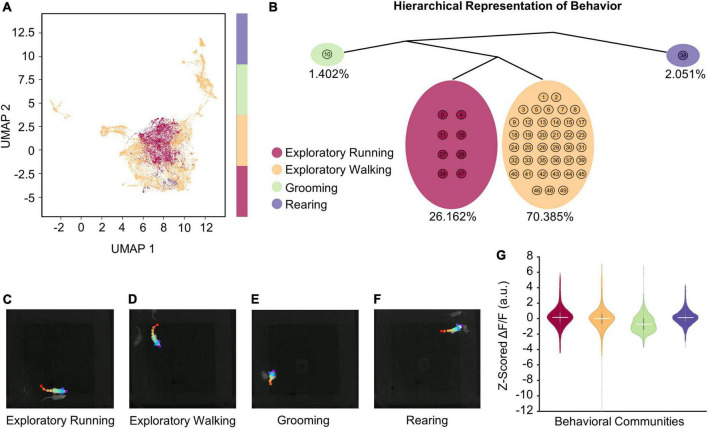
VAME reveals four distinct behaviors during open field exploration. **(A)** Unifold Manifold Approximation (UMAP) embedding of the latent representation encoded from VAME’s recurrent neural network, color-coded based on four human expert-labeled behavioral communities corresponding to exploratory running, exploratory walking, grooming, and rearing. **(B)** Hierarchical representation of the four communities of behavioral motifs in (A). Percentages below each community highlight the time duration observed for that behavior across a total of 10 h of open field exploration (*n* = 74 sessions). **(C–F)** Representative video frames for each behavioral community identified with VAME. Videos showing examples of behavioral activities for each identified community are provided in Supplementary material. **(G)** Z-scored ΔF/F associated with each behavioral community. Horizontal solid white lines indicate the medians (see Table 6 for statistics).

**TABLE 6:** Results of a linear mixed-effects model of the z-scored ΔF/F of cholinergic activity with six fixed effects, which include y-intercept, the logarithm of allocentric neck movement speed, darkness, and the behavioral communities as identified through VAME.

Fixed effects coefficients						
**Name**	**Estimate**	**SE**	**t-statistic**	**DF**	***P*-value**	**95% CI**
y-intercept	–0.240	0.021	–11.273	1,051,803	<0.0001	[–0.281 –0.198]
log_2_(speed)	0.200	0.013	14.790	1,051,803	<0.0001	[0.173 0.226]
Darkness	–0.029	0.032	–0.907	1,051,803	0.364	[–0.092 0.034]
ER	–0.001	0.042	–0.013	1,051,803	0.989	[–0.083 0.082]
Rearing	0.277	0.042	6.636	1,051,803	<0.0001	[0.195 0.359]
Grooming	–0.211	0.141	–1.495	1,051,803	0.135	[–0.488 0.066]

**Model formula**						
${\text{y}}_{\text{imjkl}}{\text{= β}}_{\text{0}}{\text{+β}}_{\text{1}}{\text{log}}_{\text{2}}\left(\text{speed}\right)\text{ +}\overset{2}{\underset{\text{m=2}}{\sum }}{\text{β}}_{\text{2m}}{\text{I[D]}}_{\text{im}}\text{+}\overset{4}{\underset{\text{j=2}}{\sum }}{\text{β}}_{\text{3j}}{\text{I[C]}}_{\text{ij}}{\text{+ b}}_{\text{0k}}{\text{A}}_{\text{k}}{\text{+ b}}_{\text{0l}}{\text{S}}_{\text{l}}{\text{+ b}}_{\text{0m}}{\text{D}}_{\text{m}}{\text{+ b}}_{\text{0j}}{\text{C}}_{\text{j}}{\text{+ ε}}_{\text{imjkl}}\text{,}$ where index *i* corresponds to the # of observations, index *m* corresponds to the darkness types, index *j* corresponds to the behavioral community types, index *k* corresponds to the animal identity, and index *l* corresponds to the session identity. *A*_*k*_ represents the *k*th animal ID, *S*_*l*_ represents the *l*th session ID, *I*[*D*]_*im*_ is the dummy variable representing level *m* of the darkness type, *I*[*C*]_*ij*_ the dummy variable representing level *j* of the behavioral community type, *b* the random mixed effects, ε_*imjkl*_ the observation error, and *y*_*imjkl*_ the response variable representing z-scored ΔF/F. Random effects and observation error are normally distributed around zero.						
**Model statistics**						
AIC: 2.761 × 10^6^	BIC: 2.761 × 10^6^	Log-likelihood: -1.4 × 10^6^	Deviance: 2.761 × 10^6^			

Random effects of the animal (n = 3) and sessions (n = 41 light and n = 32 dark) on all fixed effects were included in addition to a random error term. Total number of observations: 1,051,803; fixed effects coefficients: 6; random effects coefficients: 380; covariance parameters: 17. ER, Exploratory Running.

### Analysis software and code

Data analysis was performed using MATLAB (MathWorks) and custom-written Matlab scripts. Code will be made available upon request.

## Results

### The activity of cholinergic neurons is linearly correlated with the logarithm of the animal’s running speed

We used a recombinant adeno-associated virus (rAAV) and the Cre-loxP system to target the expression of the genetically encoded fluorescent Calcium indicator jGCaMP7s (Dana et al., 2019) to the cholinergic subpopulation in the MSDB of transgenic mice that express the Cre-recombinase under the control of the choline-acetyltransferase (ChAT) promoter (ChAT-Cre mice) (Figures 1B,C and Supplementary Figure 1). This allowed us to monitor the population activity of cholinergic neurons in the MSDB of a total of five adult ChAT-Cre mice (two females, three males; Table 1) at high temporal resolution during free foraging in an open field environment. The temporal resolution of measurements in cholinergic activity was only constrained by the kinetics of the Calcium sensor jGCaMP7s that has a reported half-decay time of 1.69-s (Dana et al., 2019), providing sufficient temporal resolution to analyze the temporal dynamics in cholinergic activity as a function of changes in the running speed of freely foraging mice. To measure the running speed of mice, we used a thermal camera (FLIR SC8000) positioned above the center of the open field box. We used the markerless pose estimation tool DeepLabCut (Mathis et al., 2018) to identify, in each video frame, the neck position of the mouse which was used to compute the running speed (Figure 1A). Consistent with a recent study using fiber photometry to monitor the release of acetylcholine in the hippocampus in freely moving mice (Zhang et al., 2021), we found that the population activity of cholinergic neurons in the MSDB was correlated to the running speed during periods of locomotion (Figure 1D). However, we were intrigued by the fact that the cholinergic activity showed the largest fluctuations at low running speeds (Figure 1E) resulting in speed tuning curves of cholinergic activity that are best fitted by a saturating exponential function, both in single sessions (see Figure 1E for one example) and when averaging data across 103 sessions from five mice (Figure 1F). Since the temporal dynamics in the cholinergic activity were not reflected in the speed tuning curves and showed larger variations at lower running speeds, we asked whether changes in cholinergic activity may correlate with changes in the *logarithm* of the running speed. In fact, cholinergic activity showed a strong and strikingly linear correlation to the logarithm of the running speed (Figures 1G–I). Theoretical work has been shown that information from optical flow could contribute to coding of location and running speed in the medial entorhinal cortex (Raudies et al., 2012, 2016). Experimental data showing the contribution of visual inputs on the spatial accuracy of grid cell firing in the medial entorhinal cortex (Chen et al., 2016; Pérez-Escobar et al., 2016; Dannenberg et al., 2020) support the idea that movement information from optical flow can contribute to speed coding in the hippocampal formation. We therefore tested the hypothesis that the relationship between the activity of cholinergic neurons in the MSDB and the animal’s running speed is changed in the absence of visual information by testing the effect of darkness on the speed tuning of cholinergic activity using a linear mixed effects model of data obtained from recordings under standard lighting conditions and darkness. Interestingly, we found no substantial change in the speed tuning of cholinergic activity during darkness (Figures 1J,K and Table 2). These data indicate that, during free foraging in an open field box, the temporal dynamics in the activity of cholinergic neurons in the MSDB are primarily a function of the animal’s movement speed with a negligible influence of visual cues.

### The activity of cholinergic neurons is linearly correlated to neck movements during stationary behavior

We noticed that cholinergic activity showed huge variations for running speeds below 5–10 cm/s. Our previous analysis primarily focused on the analysis of locomotion as opposed to other characteristic behaviors or body movements that are not related to locomotion but can also occur during stationary periods, i.e., in the absence of locomotion. However, the observed large variance in cholinergic activity for small running speeds below 3-cm/s and the fact that the observed linear correlation of cholinergic activity with the logarithm of the animal’s running speed holds true even for very small running speeds suggest that cholinergic activity might also be correlated to neck movements while the mouse is stationary in space (Figure 1I). To make sure that our analysis of neck movements during stationary periods was not confounded by the effects of stop-and-go-patterns during locomotion, we analyzed a subset of *n* = 16 out of 103 sessions in which the mouse was stationary (allocentric neck movement speed < 3-cm/s) for more than two thirds of the total length of the recording session (Figure 2). Intriguingly, we found that the strong and linear correlation of cholinergic activity with the logarithm of the animal’s running speed extended to the logarithm of the animals’ neck movement speed during stationary activity. To test whether the speed tuning of cholinergic activity during stationary activity depends on visual cues or optic flow, we again modeled the effect of darkness on speed tuning during stationary activity using a linear mixed-effects model (Table 3). As for the speed tuning during locomotion, we found no significant effect of darkness on speed tuning of cholinergic modulation during stationary activity. In summary, these data suggest that a potential speed code by the population activity of cholinergic MSDB neurons does not distinguish between stationary periods and locomotion but instead codes for the whole range of translational neck movement speeds in the horizontal plane between 0-cm/s and the animal’s maximal running speed.

### Temporal dynamics in cholinergic activity are fast enough to match changes in movement speed

Speed tuning curves of cholinergic activity do not provide any information about the temporal dynamics in cholinergic modulation. If changes in the population activity of cholinergic neurons are fast enough to align with changes in the speed of neck movements during stationary activity or running, the population activity of cholinergic neurons could potentially be used in computational models to provide a movement speed signal in real-time. To quantify the temporal dynamics in cholinergic modulation, we computed the integration time window for cholinergic activity that maximized the correlation between cholinergic activity and the logarithm of the neck movement speed of an animal. When averaging across 103 sessions from five mice, we found that an integration time window of just 1.3-s optimized the correlation between the population activity of cholinergic MSDB neurons and the animals’ running speed during locomotion or neck movement speeds during stationary activity (Figure 3A). Notably, 1.3-s is shorter than the reported half-decay time of jGCaMP7s, the fluorescent Calcium sensor used in this study to monitor the activity of cholinergic MSDB neurons, indicating that our time scale estimate of changes in cholinergic activity reached the temporal resolution limit of what we could detect in this study. We conclude that changes in MSDB cholinergic activity are fast enough to match changes in running speed during locomotion or neck movements during stationary behavior and that changes in cholinergic activity could potentially be even faster than we were able to detect in this study. Optimal integration time windows that maximize the correlation of the smoothed cholinergic activity and the neck movement speed of the animal were computed for each session. This allowed us to analyze the distribution of optimal integration windows across sessions (Figure 3B). We found that the distribution is quite narrow with a strong peak between 0.8 and 1 s, further validating our previous finding that changes in cholinergic activity are fast and align well with the time scale of changes in the animal’s running speed during locomotion or neck movement speeds during stationary behavior. We next tested whether the temporal alignment of cholinergic activity with locomotion or neck movements during stationary activity depends on visual inputs or optic flow by comparing data during standard room lighting and darkness sessions (light: *n* = 55 sessions, five mice; dark: *n* = 47 sessions, four mice). Consistent with the results of our previous analysis of speed tuning curves of cholinergic activity during darkness, we found that the relationship between changes in cholinergic activity and changes in the animal’s running speed or neck movement speeds during stationary activity remained unaltered during darkness (Figures 3C,D); there was no significant difference between the Pearson’s correlation coefficients for the correlation between 1.3 s smoothed cholinergic activity and the logarithm of movement speed between sessions recorded during standard lighting and darkness (R_Light_ = 0.46 ± 0.17; R_Dark_ = 0.46 ± 0.13; *t*(100) = -0.192, *p* = 0.85, 95% CI = [-0.07 0.05]). Likewise, the distribution of optimal integration windows computed for each session were not significantly different between standard lighting and darkness conditions (*p* = 0.29, Kolmogorov-Smirnov test). Next, we asked whether the temporal dynamics in cholinergic activity during stationary activity align with changes in the speed of an animal’s neck movements on a similarly fast time scale as they do with changes in running speed during locomotion. To answer this question, we analyzed the subset of *n* = 16 sessions in which the mice remained stationary (allocentric neck movement speed < 3-cm/s) for more than two thirds of the session time. We found that the integration time window that maximizes the correlation of cholinergic activity and the neck movement speed during stationary activities was 1.7 s, thus not substantially different from the integration time window of 1.3-s that maximized the correlation of cholinergic activity in the complete data set (Figures 3E,F). We next asked whether information on movement speed helps predict cholinergic activity or vice versa. To address this question, we used Granger causality analysis (Granger, 1969; Geweke, 1982, 1984), a statistical notion of causality applicable to time series data, whereby cause precedes, and helps predict, effect (Barnett and Seth, 2014). The results of this analysis show that the logarithm of movement speed helps predict, i.e., “Granger-causes,” cholinergic activity and vice versa (see Table 4 for statistics); in other words, information about movement speed helps predict cholinergic activity and information about cholinergic activity helps predict movement speed. However, in three out of five animals, the magnitude of Granger causality was significantly higher in the direction from movement speed to cholinergic activity than in the opposite direction (Figures 3G–I; see Table 5 and Supplementary Table 1 for statistics). No such difference was found in the remaining two animals. Taken together, these data demonstrate that the movement speed of an animal informs the activity of MSDB cholinergic neurons and vice versa.

### Cholinergic neurons in the medial septum/diagonal band of Broca are more active during rearing

The data presented so far indicate a strong correlation between cholinergic MSDB activity and the logarithm of the neck movement speed of an animal, regardless of whether those movements are caused by locomotion, i.e., translational movements due to walking or running, or by stationary activity during characteristic behaviors such as grooming or rearing. However, a recent study has shown that cholinergic activity is low during the occurrence of sharp-wave-ripples in the hippocampus, 40–100 ms long events of synchronous network activity detected in the local field potential that typically occur while the mouse is stationary (Zhang et al., 2021). Conversely, rearing, a stereotypic behavior that is associated with attention, exploration, and integration of sensory inputs, has been shown to be accompanied by an increase in the frequency of theta rhythmic activity and theta-gamma coupling (Barth et al., 2018), hippocampal network states that are typically associated with higher cholinergic activity. However, it is unclear whether changes in cholinergic activity associated with characteristic behaviors such as grooming or rearing are driven by changes in internal or cognitive states or by associated changes in movement speed. To distinguish between these two possibilities, we asked whether engagement in a characteristic behavior affects the activity of cholinergic MSDB neurons beyond what is expected from the change in movement speed associated with that behavior. To answer this question, we first chose an unbiased approach to identify discrete behavioral clusters using the deep learning tool “VAME” (variational animal motion embedding) (Luxem et al., 2022). VAME takes the body part positions identified by DeepLabCut as input to train a model of animal motion that is then used to classify distinct motion patterns into clusters of similar behavioral activities. To improve the accuracy of VAME, we increased the number of DeepLabCut labels from six to eleven and relabeled the videos from *n* = 74 sessions of three mice where the videos were taken with the same camera and video settings to ensure robust model performance across sessions. VAME identified four main clusters (“communities”) of behavioral activities (Figures 4A,B) that could be identified *post hoc* by the experimenter as “exploratory running,” “exploratory walking,” “grooming,” and “rearing” (Figures 4C–F and Supplementary Videos 1–4). On average, mice spent 1.4% of their time grooming, 26.2% of their time walking, 70.4% of their time running, and 2.1% of their time rearing.

To correct for expected effects of movement speed on cholinergic activity, we used a linear mixed effects model to test the movement speed-corrected effect of the four identified main behavioral clusters on the activity of cholinergic neurons in the MSDB. After correcting for movement speed, we found a significant positive effect of rearing on cholinergic activity, while in the absence of movement speed effects, the additional behavioral aspects of exploratory walking, exploratory running, and grooming had no significant impact on the activity of cholinergic neurons (Table 6; see also Figure 4G, Supplementary Figure 2, and Supplementary Tables 2, 3). Furthermore, we found a highly significant effect of the logarithm of the neck movement speed on cholinergic activity but no significant effect of darkness on cholinergic activity (Table 6).

Taken together, these data demonstrate that cholinergic neurons in the MSDB are more active during rearing as compared to other characteristic behaviors such as grooming, exploratory walking, or exploratory running when corrected for effects of movement speed.

## Discussion

Experiments and analyses presented in this study investigate the temporal dynamics of cholinergic activity in the septo-hippocampal system in freely foraging mice as a function of the animal’s running speed during locomotion, the speed of neck movements during stationary activity, visual inputs, and discrete behavioral motifs identified by unsupervised VAME. The presented results demonstrate that the temporal dynamics in the population activity of MSDB cholinergic neurons are fast enough to align with the temporal dynamics of running speed during locomotion periods as well as temporal dynamics in the speed of neck movements during periods of stationary activity such as grooming or stationary head movements. Intriguingly, the logarithm of the speed of neck movements correlated strongly and linearly across the whole range of speed values between 0-cm/s and the maximal running speed with no detectable change in the speed tuning of cholinergic activity at the transition point from stationary activity to locomotion. Furthermore, the quantification of temporal dynamics in the cholinergic activity and their relationship to the speed of the animal revealed that cholinergic neurons can change their activity fast enough to match changes in running speed. Notably, no differences in cholinergic activity and its relationship to running speed or neck movements during stationary activity were detected during darkness. Lastly, an analysis adjusted for effects of movement speed of the effect of four discrete clusters of behavioral activity that correspond to exploratory running, exploratory walking, grooming, and rearing revealed that rearing is associated with higher cholinergic activity than expected from the movement speed during rearing alone.

Importantly, cholinergic modulation can act on different time scales ranging from milliseconds to minutes (transient or tonic, fast, or slow) with important consequences on cortical dynamics. However, the temporal dynamics in cholinergic activity, particularly in relation to running speed, have not been quantified so far, mostly due to a lack of recording techniques that allowed the recording of cholinergic activity in the MSDB at fast temporal resolution in freely behaving mice. Previous measurements of cholinergic activity in the septo-hippocampal system used microdialysis (Marrosu et al., 1995) or amperometry (Parikh et al., 2007; Teles-Grilo Ruivo et al., 2017) techniques to measure changes in the release of acetylcholine at a temporal resolution of minutes or multiple seconds, respectively. In this study, we used a fiber photometry approach to monitor the population activity of cholinergic neurons in the MSDB on a time scale of ∼1-s that proved fast enough to study the temporal dynamics in relation to changes in movement speed. Cholinergic projection neurons in the MSDB are the primary and major source of cholinergic innervation of the hippocampal formation (Mesulam et al., 1983; Rye et al., 1984) and have a key function in modulating hippocampal activity *via* a direct or indirect pathway (Alreja et al., 2000; Dannenberg et al., 2015). In addition to cholinergic neurons, the MSDB contains glutamatergic and GABAergic neurons. All three cell types form an interconnected network within the MSDB and project to the hippocampal formation (Robinson et al., 2016; Müller and Remy, 2017). While the MSDB as a whole is important for modulating hippocampal network functions related to memory, spatial cognition, and memory-guided navigation, the population of cholinergic neurons in the MSDB is of particular interest because the neurotransmitter acetylcholine is an important neuromodulator of cognitive functions and behavior. Importantly, acetylcholine can modulate hippocampal network functions *via* binding to nicotinic and muscarinic receptors with different time courses of effect. Moreover, the activity of cholinergic MSDB neurons can modulate hippocampal network functions indirectly *via* recruiting glutamatergic and GABAergic MSDB projection neurons (Alreja et al., 2000). Experimental data show that optogenetic activation of glutamatergic MSDB neurons initiated movements in mice (Fuhrmann et al., 2015; Robinson et al., 2016) and optogenetic activation of GABAergic neurons modulated theta rhythmic activity (Dannenberg et al., 2015; Zutshi et al., 2018) that is correlated to the running speed of an animal. Because of cholinergic effects in the medial septum, fiber-photometric recordings of the activity of cholinergic projection neurons in the MSDB allows a more holistic interpretation of the temporal dynamics in cholinergic activity compared to data on the synaptic release of acetylcholine in the hippocampal formation. Future studies analyzing and comparing the temporal dynamics in synaptic release of acetylcholine in the hippocampal formation will need to determine whether there are differences in the temporal dynamics of the population activity of MSDB cholinergic neurons and the temporal dynamics in the synaptic release of acetylcholine in the hippocampus and related structures.

The fast temporal dynamics in conjunction with the strong and linear correlation of cholinergic activity with the logarithm of the animal’s running speed during locomotion support the hypothesis that the activity of cholinergic neurons in the MSDB can provide a speed signal to the hippocampus and medial entorhinal cortex. Moreover, Granger causality analysis revealed that information about movement speed helps predict cholinergic activity suggesting that the activity of cholinergic neurons change in response to changes in movement speed. The finding that the temporal dynamics in cholinergic activity and its correlation to running speed are unaltered during darkness provides further support for the hypothesis that a cholinergic speed signal could be used for path integration. Notably, the Granger causality analysis did not only show that movement speed helps predict cholinergic activity but also the opposite, namely that cholinergic activity helps predict the movement speed of the animal. This result is consistent with the known roles of cholinergic neuro-modulatory function in the context of arousal, attention, and memory (Marrosu et al., 1995; Hasselmo and McGaughy, 2004; Hasselmo, 2006) and with the fact that a change in cognitive state often causes a change in physical activity.

Computational models of path integration typically rely on a linear speed signal. Speed cells in the medial entorhinal cortex have been suggested to provide a code for running speed (Kropff et al., 2015; Hinman et al., 2016; Carvalho et al., 2020). However, the temporal dynamics in speed cells’ firing rates below 1-s have been shown to not accurately match changes in the running speed of animals (Góis and Tort, 2018; Dannenberg et al., 2019). Data presented in this study suggest that cholinergic modulation could provide a speed signal on the time scale of 1-s used by a neural reader mechanism (Buzsáki, 2010) in the hippocampus and entorhinal cortex. Importantly, the signal is linearly correlated to the logarithm of the animal’s movement speed and extends into stationary periods. The latter is a potential advantage over other proposed speed codes such as the speed code by entorhinal speed cells (Kropff et al., 2015; Ye et al., 2018) and a proposed speed signal represented by theta rhythmic activity in the local field potential or by theta rhythmic spiking of neurons (Hinman et al., 2016). In previous studies, the speed signal represented by firing rates of neurons in the medial entorhinal cortex was only tested on running speeds above a speed threshold of 3-cm/s. Likewise, a potential speed code by theta frequency or theta amplitude is restricted to periods of locomotion due to the absence of theta rhythmic activity during most stationary activities such as grooming. However, the speed of head movements or the speed of changes in body position is likely an important factor for path integration, even if those movements occur while the mouse remains stationary.

Data presented in this study provide experimental evidence that cholinergic MSDB neurons are an important component of neural circuits that control or code for running speed. Since fiber photometry measures the bulk activity of many neurons, data presented in this study demonstrate a correlation between the population activity of cholinergic MSDB neurons and movement speed. Since cholinergic projection neurons broadly innervate many different subregions in the hippocampal formation and can influence network activity, a cholinergic signal would be ideally suited to transmit a speed signal that modulates the network activity of multiple target regions. Previous studies have shown that lesions of cholinergic projection neurons impair path integration (Martin and Wallace, 2007; Martin et al., 2007; Hamlin et al., 2013; Yoder et al., 2017) providing experimental support that cholinergic activity could be used as a speed signal by the hippocampus and medial entorhinal cortex. An alternative—not mutually exclusive—interpretation of the current data is that the activity of MSDB cholinergic neurons is primarily correlated to physical activity instead of the logarithm of movement speed. This interpretation is supported by the fact that cholinergic activity remains strongly and linearly correlated to the logarithm of the speed of neck movements during stationary activity. Future experiments need to address questions related to the collinearity of movement speed and physical activity.

Experimental data from the literature suggest that cholinergic activity is modulated by behavioral activity and internal brain states or cognition, in addition to movement speed. In fact, despite the strong correlation between the logarithm of movement speed and activity of cholinergic MSDB neurons observed in this study, the variability in the cholinergic signal cannot be fully explained by changes in movement speed alone. This variability that is unexplained by movement speed may stem from the diverse roles and functions of cholinergic activity described in the literature for a wide variety of cognitive processes, such as sensory integration and cue detection (Pinto et al., 2013; Gritton et al., 2016), stress responses, novelty, encoding of memories, and attention and arousal (Marrosu et al., 1995; Hasselmo and McGaughy, 2004; Hasselmo, 2006; Wallace et al., 2011; Higley and Picciotto, 2014; Harrison et al., 2016). One possible explanation for the unexplained variability in cholinergic activity is that temporal dynamics in cholinergic activity might be a function of visual inputs or optic flow. An alternative hypothesis discussed in the next paragraph is that cholinergic activity is modulated by behavioral activity beyond the correlation to movement speed. The first hypothesis is supported by experimental data showing that cholinergic activity is significantly correlated with cue detection (Gritton et al., 2016) and visual perception (Goard and Dan, 2009; Niell and Stryker, 2010; Pinto et al., 2013) and attention (Hasselmo and McGaughy, 2004). We therefore tested whether the observed temporal dynamics in cholinergic activity and their relationship to running speed change during darkness. Interestingly, the relationship of cholinergic dynamics to changes in running speed remained unaltered during locomotion in darkness. Likewise, the relationship of cholinergic dynamics to changes in the speed of the animal’s neck movements during darkness appeared unaltered during stationary activity. These findings are consistent with findings of a previous study showing that glutamatergic cells in the MSDB remain tuned to running speed in the absence of optic flow (Justus et al., 2016). The finding that visual inputs do not alter the temporal dynamics of cholinergic activity as a function of running speed are similar to findings in previous studies of a potential speed code by firing rate in neurons of the entorhinal cortex (Dannenberg et al., 2020), in retrosplenial cortex (Carstensen et al., 2021), and posterior parietal cortex (Alexander et al., 2022) that demonstrated no change in the speed tuning of normalized firing rates during darkness. However, those studies showed a change in the *gain* of speed tuning of absolute firing rates during darkness. Since the analysis of fiber-photometry data requires the normalization of cholinergic activity, we cannot compare absolute values of cholinergic activity across sessions. It thus remains to be tested whether the absence of visual inputs can change baseline levels of cholinergic activity or the gain in the speed tuning of cholinergic activity.

The second hypothesis mentioned in the previous paragraph, namely that population activity of cholinergic MSDB neurons is modulated by behavioral or cognitive state, is supported by experimental data showing that acetylcholine release in the hippocampal formation is correlated to active exploration and learning and memory (Marrosu et al., 1995; Hasselmo, 2006). Empirical data suggest that rearing, a behavioral action in a stationary location where mice stand on their hindlimbs to scan the environment from an elevated perspective, is associated with a distinct brain state supporting the encoding of spatial information from distant visual cues (Barth et al., 2018). Interestingly, we found that rearing was correlated with higher cholinergic activity; however, this correlation did not depend on the presence of visual cues (no significant interaction effect between rearing and darkness on cholinergic activity; *p*-value = 0.290; linear mixed-effects model; see Supplementary Table 2). One explanation is that other sensory modalities are important, in particular when the mouse is rearing with the support of the walls of the environment, as frequently observed in our data (see video showing examples of rearing behavior in Supplementary material). These data are consistent with the role of acetylcholine for processing sensory information and the encoding of memories (Hasselmo, 2006). However, there is an alternative explanation that deserves to be considered. During rearing, the mouse moves vertically instead of horizontally. Due to the centered position of the camera used for the tracking of mice, vertical movements cannot be detected. Future studies need to address the question whether the observed increase in cholinergic activity during rearing is primarily due to the cognitive demand of processing sensory information or encoding novel memories, or due to coding the movement speed in the vertical axis.

## Data availability statement

The raw data supporting the conclusions of this article will be made available by the authors, without undue reservation.

## Ethics statement

The animal study was reviewed and approved by the Institutional Animal Care and Use Committee for the Charles River Campus at Boston University.

## Author contributions

HD and MH designed the research. HD, HL, and PN conducted the experiments. HD, JK, and KH analyzed the data. HD, JK, and MH wrote and revised the manuscript. All authors contributed to the article and approved the submitted version.

## Supplementary material

Supplementary Figure 1 Visualization of fiber track position and colocalization of jGCaMP7s and ChAT immunostaining. Example images are shown for three mice. Each row shows data on medial septal slices from one mouse. Magenta colors indicate positive immunolabeling for choline acetyltransferase (ChAT), a marker for cholinergic neurons; green colors indicate presence of jGCaMP7s. Note that jGCaMP7s expression is largely confined to ChAT-positive cholinergic neurons (78 ± 6% jGCaMP7s-positive neurons were co-labeled for ChAT; mean ± s.e.m; *n* = 4 mice; total of 437 cells from 1 to 3 slices per mouse; note that this is likely an underestimation because cells showed signs of cell death due to overexpression of jGCaMP7s). The same images provide histological verification that the optical fiber tip was located within the medial septum.

Supplementary Figure 2 Differences in movement speed across behavioral clusters. Violin plots show the distribution of movement speeds associated with each behavioral cluster during open field exploration identified by VAME. Boxplots within violin plots indicate the 25th–75th percentiles. Horizontal solid white lines indicate the medians of the distributions.
