# Supplementary material for: Temporal dynamics of cholinergic activity in the septo-hippocampal system
Source: Front Neural Circuits. 2022 Aug 25;16:957441. doi: 10.3389/fncir.2022.957441 (PMC9452968; doi:10.3389/fncir.2022.957441)
Supplement: Supplementary Figure 1 — Visualization of fiber track position and colocalization of jGCaMP7s and ChAT immunostaining. Example images are shown for three mice. Each row shows data on medial septal slices from one mouse. Magenta colors indicate positive immunolabeling for choline acetyltransferase (ChAT), a marker for cholinergic neurons; green colors indicate presence of jGCaMP7s. Note that jGCaMP7s expression is largely confined to ChAT-positive cholinergic neurons (78 ± 6% jGCaMP7s-positive neurons were co-labeled for ChAT; mean ± s.e.m; n = 4 mice; total of 437 cells from 1 to 3 slices per mouse; note that this is likely an underestimation because cells showed signs of cell death due to overexpression of jGCaMP7s). The same images provide histological verification that the optical fiber tip was located within the medial septum. [file Data_Sheet_2.docx]

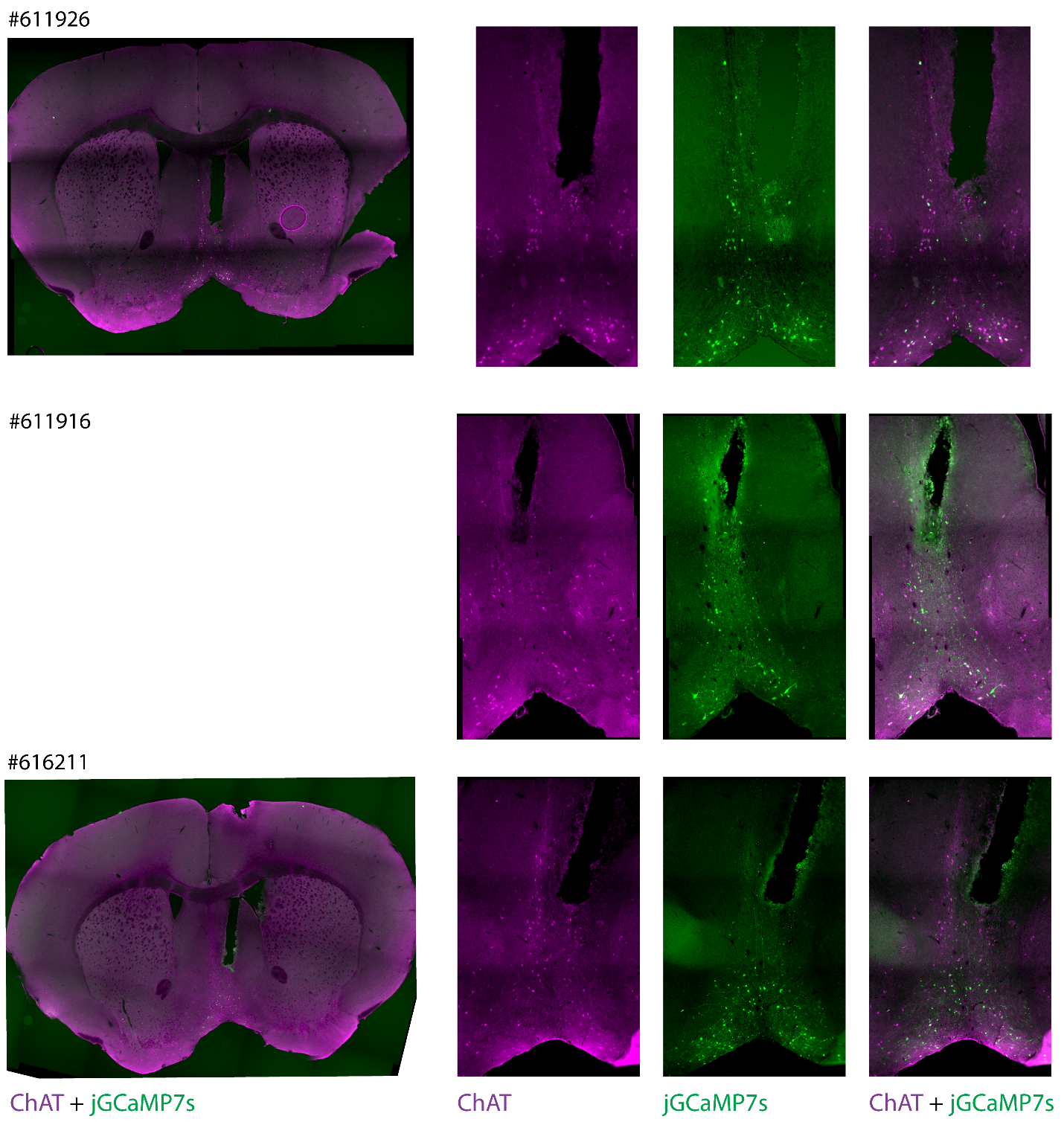


**Supplementary Figure S1 related to Figure 1: Visualization of fiber track position and colocalization of jGCaMP7s and ChAT immunostaining.** Example images are shown for three mice. Each row shows data on medial septal slices from one mouse. Magenta colors indicate positive immunolabeling for choline acetyltransferase (ChAT), a marker for cholinergic neurons; green colors indicate presence of jGCaMP7s. Note that jGCaMP7s expression is largely confined to ChAT-positive cholinergic neurons (78% ± 6% jGCaMP7s-positive neurons were co-labeled for ChAT; mean ± s.e.m; n = 4 mice; total of 437 cells from 1–3 slices per mouse; note that this is likely an underestimation because cells showed signs of cell death due to overexpression of jGCaMP7s). The same images provide histological verification that the optical fiber tip was located within the medial septum.


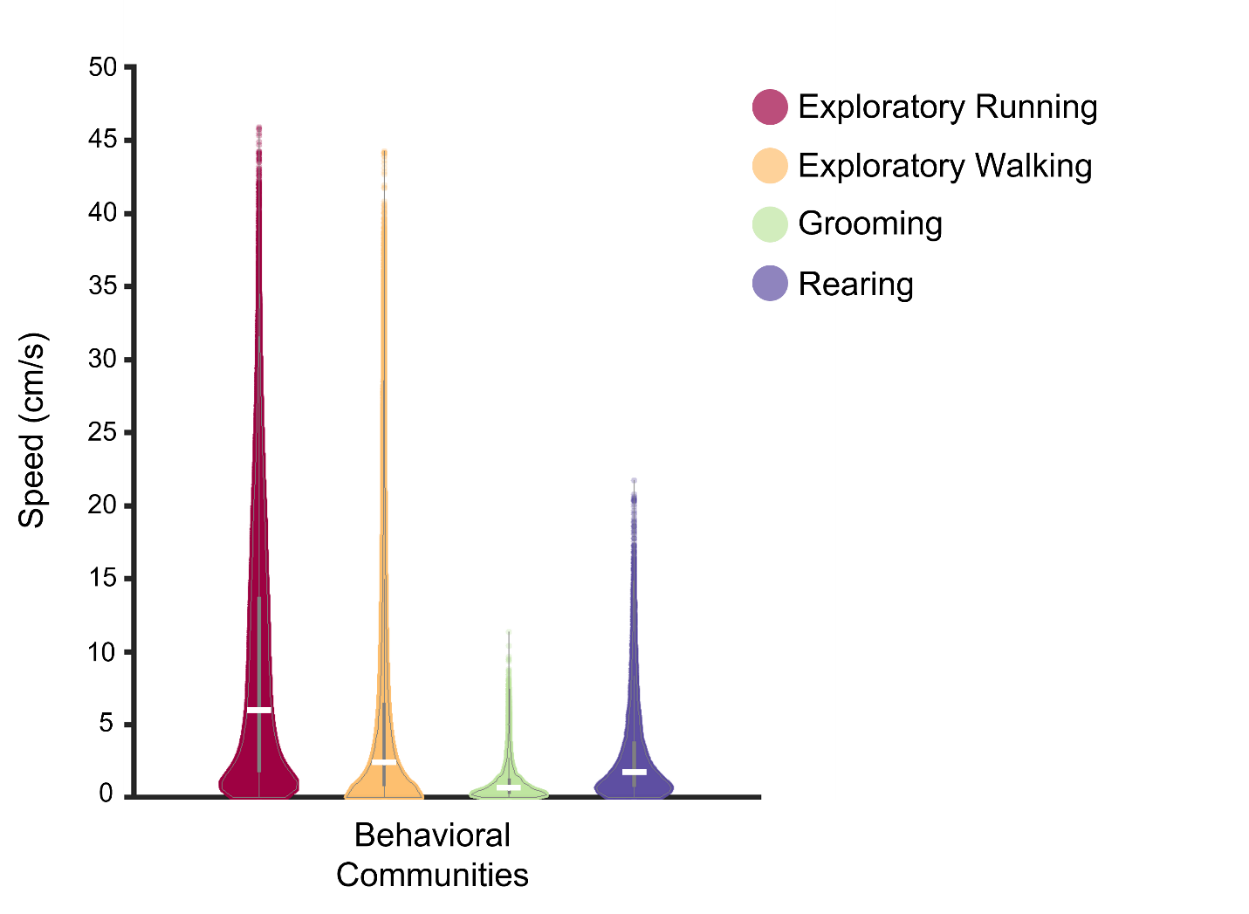


**Supplementary Figure S2: Running speeds differ across behavioral clusters.** Violin plots show the distribution of movement speeds associated with each behavioral cluster during open field exploration identified by VAME. Boxplots within violin plots indicate the 25^th^ – 75^th^ percentiles; horizontal solid white lines indicate the medians of the distributions.
