# Supplementary material for: Temporal dynamics of cholinergic activity in the septo-hippocampal system
Source: Front Neural Circuits. 2022 Aug 25;16:957441. doi: 10.3389/fncir.2022.957441 (PMC9452968; doi:10.3389/fncir.2022.957441)
Supplement: Supplementary file 2 [file Table_1.DOCX]

| **Mouse** | **Name** | **Estimate** | **SE** | **t-statistic** | | **DF** | **p-value** | **95% CI** |
| --- | --- | --- | --- | --- | --- | --- | --- | --- |
| #611915 | ACh🡪Speed | 0.0011 | 0.00067 | | 1.700 | 36 | 0.097793 | [–0.00022 0.00249] |
| #611916 | ACh🡪Speed | –0.0003 | 0.00077 | | –0.323 | 86 | 0.74718 | [–0.00178 0.00128] |
| #611926 | ACh🡪Speed | –0.0020 | 0.00068 | | –3.007 | 14 | 0.009425 | [–0.00348 –0.00058] |
| #616195 | ACh🡪Speed | –0.0055 | 0.00123 | | –4.431 | 30 | 0.000116 | [–0.00798 –0.00295] |
| #616211 | ACh🡪Speed | –0.0048 | 0.00096 | | –5.038 | 20 | 6.30x10^-05^ | [–0.00685 –0.00284] |

**Supplementary Table S1**. Results of a linear mixed-effects model testing the direction of Granger causality on the magnitude of Granger causality in each mouse. The linear mixed-effects model formula is the same as the one reported in Table 4. See Table 1 for information on sessions per animal. In three animals, namely #611926, #616195, and #616211, Granger causality magnitude is significantly smaller in the direction from cholinergic activity (ACh) to movement speed (Speed) compared to the opposite direction.

| **Fixed effects coefficients** | | | | | | | |
| --- | --- | --- | --- | --- | --- | --- | --- |
| Name | Estimate | SE | t-statistic | DF | p-value | 95% CI |  |
| Intercept | –0.240 | 0.021 | –11.268 | 1051799 | <0.0001 | [–0.281 –0.198] |  |
| $\log_{2}\left( \mathrm{speed} \right)$ | 0.200 | 0.015 | 13.461 | 1051799 | <0.0001 | [0.171 0.229] |  |
| Darkness | –0.029 | 0.032 | –0.907 | 1051799 | 0.365 | [–0.092 0.034] |  |
| ER | –0.015 | 0.054 | –0.285 | 1051799 | 0.776 | [–0.121 0.090] |  |
| Rearing | 0.310 | 0.048 | 6.472 | 1051799 | <0.0001 | [0.216 0.404] |  |
| Grooming | –0.241 | 0.174 | –1.391 | 1051799 | 0.164 | [–0.582 0.098] |  |
| $\log_{2}\left( \mathrm{speed} \right)$*Darkness | 0.000 | 0.016 | –0.023 | 1051799 | 0.982 | [–0.032 0.031] |  |
| ER*Darkness | 0.054 | 0.042 | 1.279 | 1051799 | 0.201 | [–0.029 0.137] |  |
| Rearing*Darkness | –0.074 | 0.070 | –1.058 | 1051799 | 0.290 | [–0.211 0.063] |  |
| Grooming*Darkness | –0.005 | 0.154 | –0.033 | 1051799 | 0.974 | [–0.307 0.297] |  |

| Model Formula |
| --- |
| $y_{imjkl}= \beta_{0}+{\beta_{1}log}_{2}\left( speed \right) + \sum_{m=2}^{2} \beta_{2m}I{[D]}_{im}+ \sum_{m=2}^{2} \beta_{3m}{log}_{2}\left( speed \right)*I{[D]}_{im}+ \sum_{j=2}^{4} \beta_{4j}I{[C]}_{ij}+ \sum_{m=2}^{2} \sum_{j=2}^{4} \beta_{5mj}I{[D]}_{im}*I{[C]}_{ij}+b_{0k}A_{k}+ b_{0l}S_{l}+ b_{0m}D_{m}+ b_{0j}C_{j}+ b_{0jm}{(C*D)}_{jm}+\varepsilon_{imjkl},$ where index *i* corresponds to the # of observations, index *m* corresponds to the darkness types, index *j* corresponds to the behavioral community types, index *k* corresponds to the animal ID, and index *l* corresponds to the session ID. $A_{k}$ represents the *k*^th^ animal ID, $S_{l}$ represents the *l*^th^ session ID, $I{[D]}_{im}$ is the dummy variable representing level *m* of the darkness type, $I{[C]}_{ij}$ the dummy variable representing level *j* of the behavioral community type, ${(C*D)}_{jm}$ represents the *j*^th^ behavioral community type nested in the *m*^th^ darkness type, *b* the random mixed effects, $\varepsilon_{imjkl}$ the observation error, and $y_{imjkl}$ the response variable representing z-scored ΔF/F. |

| **Model Statistics** | | | |
| --- | --- | --- | --- |
| AIC: 2761020 | BIC: 2761507 | Log-likelihood: -1380469 | Deviance: 2760938 |

**Supplementary Table S2.** Results of a linear mixed-effects model of the z-scored ΔF/F of cholinergic activity with ten fixed effects, which include y-intercept, the logarithm of allocentric neck movement speed, darkness, the interaction between the logarithm of allocentric neck movement speed and darkness, the behavioral communities as identified through VAME, and the interaction between the behavioral communities and darkness. Random effects of the animal (n = 3) and sessions (n = 41 light and n = 32 dark) on all fixed effects were included in addition to a random error term. Total number of observations: 1051799; fixed effects coefficients: 10; random effects coefficients: 684; covariance parameters: 31. ER, Exploratory Running.

| Fixed effects coefficients | | | | | | | | | | |
| --- | --- | --- | --- | --- | --- | --- | --- | --- | --- | --- |
| Name | | Estimate | SE | | t-statistic | DF | P | | CI |  |
| Intercept | | –0.242 | 0.020 | | –12.020 | 1051805 | <0.0001 | | [–0.282 –0.203] |  |
| $\log_{2}\left( \mathrm{speed} \right)$ | | 0.201 | 0.018 | | 10.941 | 1051805 | <0.0001 | | [0.165 0.236] |  |
| Darkness | | –0.037 | 0.030 | | –1.215 | 1051805 | 0.225 | | [–0.097 0.023] |  |
| $\log_{2}\left( \mathrm{speed} \right)$*Darkness | | 0.005 | 0.016 | | 0.317 | 1051805 | 0.752 | | [–0.027 0.038] |  |
| Model Formula | | | | | | | | | | |
| $y_{imjk}= \beta_{0}+{\beta_{1}log}_{2}\left( speed \right) + \sum_{m=2}^{2} \beta_{2m}I{[D]}_{im}+ \sum_{m=2}^{2} \beta_{3m}{log}_{2}\left( speed \right)*I{[D]}_{im}+b_{0j}A_{j}+ b_{0k}S_{k}+ b_{0m}D_{m}+\varepsilon_{imjk},$ where index *i* corresponds to the # of observations, index *m* corresponds to the darkness types, index *j* corresponds to the animal ID, and index *k* corresponds to the session ID. $A_{k}$ represents the *k*^th^ animal ID, $S_{l}$ represents the *l*^th^ session ID, $I{[D]}_{im}$ is the dummy variable representing level *m* of the darkness type, *b* the random mixed effects, $\varepsilon_{imjk}$ the observation error, and $y_{imjk}$ the response variable representing z-scored ΔF/F. | | | | | | | | | | |
| Model statistics | | | | | | | | | | |
| AIC: 2776339 | BIC: 2776469 | | | Log-likelihood: -1388158 | | | | Deviance: 2776317 | | |

**Supplementary Table S3.** Results of a linear mixed-effects model of the z-scored ΔF/F of cholinergic activity with four fixed effects, which include y-intercept, the logarithm of allocentric neck movement speed, darkness, and the interaction between the logarithm of allocentric neck movement speed and darkness. Random effects of the animal (n = 3) and sessions (n = 41 light and n = 32 dark) on all fixed effects were included in addition to a random error term. Total number of observations: 1051805; fixed effects coefficients: 4; random effects coefficients: 228; covariance parameters: 7.
