# Supplementary material for: Temporal dynamics of cholinergic activity in the septo-hippocampal system
Source: Front Neural Circuits. 2022 Aug 25;16:957441. doi: 10.3389/fncir.2022.957441 (PMC9452968; doi:10.3389/fncir.2022.957441)

Mouse #611915
Session ID: 210428_Rec1
Lighting: Light

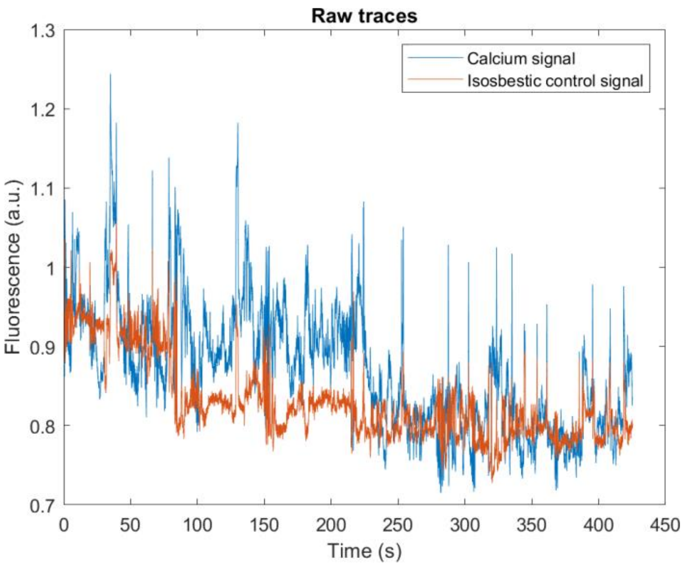

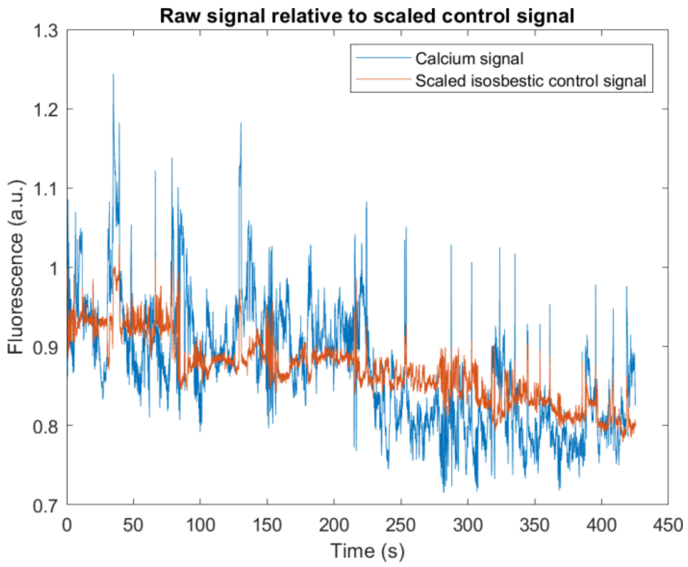

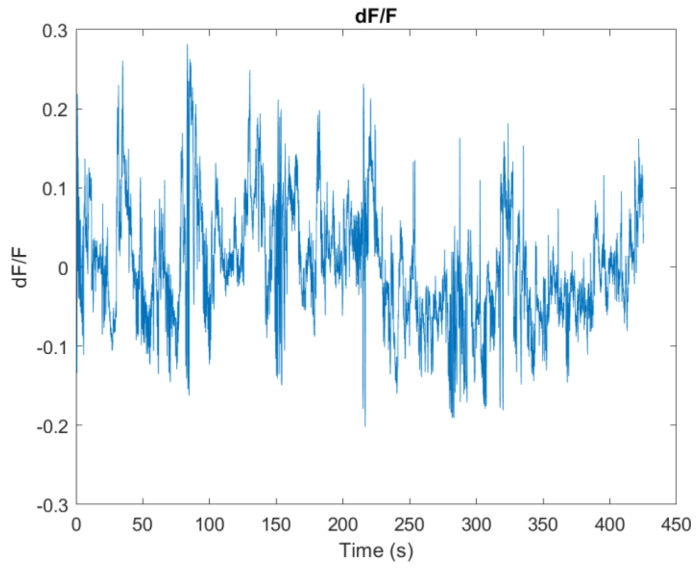

Mouse #611915
Session ID: 210428_Rec2
Lighting: Dark

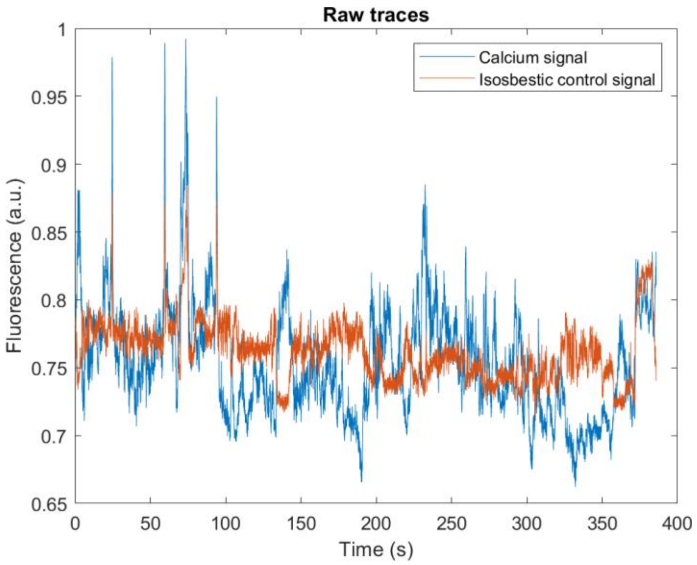

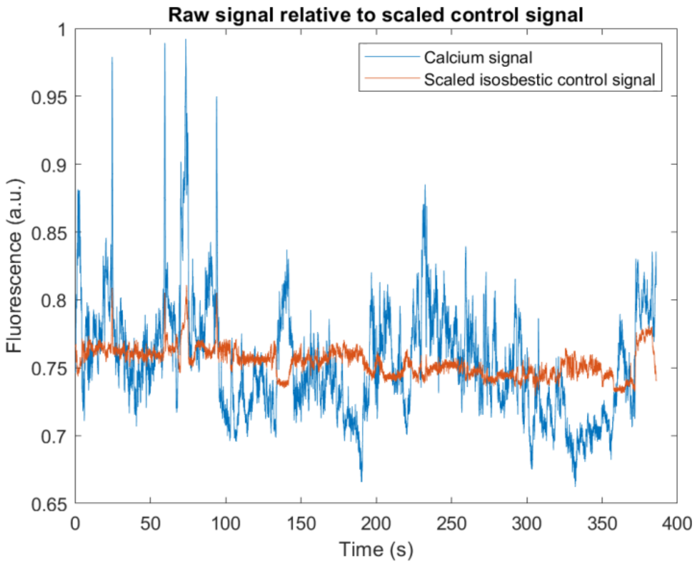

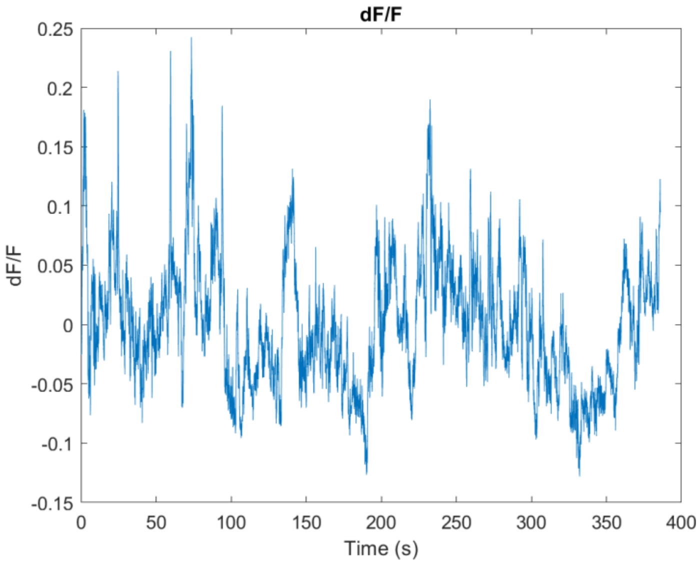

Mouse #611916
Session ID: 210506_Rec4
Lighting: Light

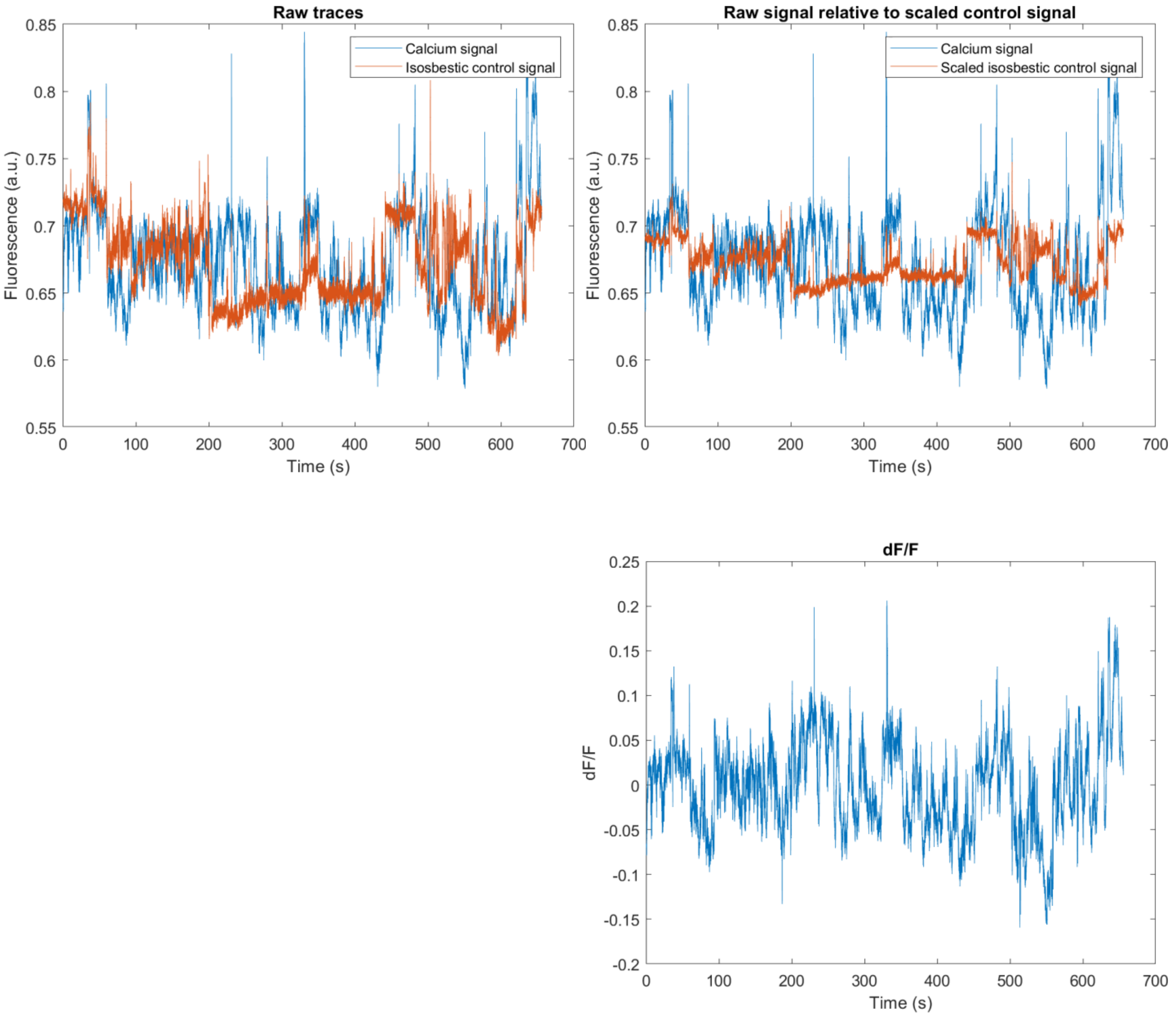

Mouse #611916
Session ID: 210505_Rec3
Lighting: Dark

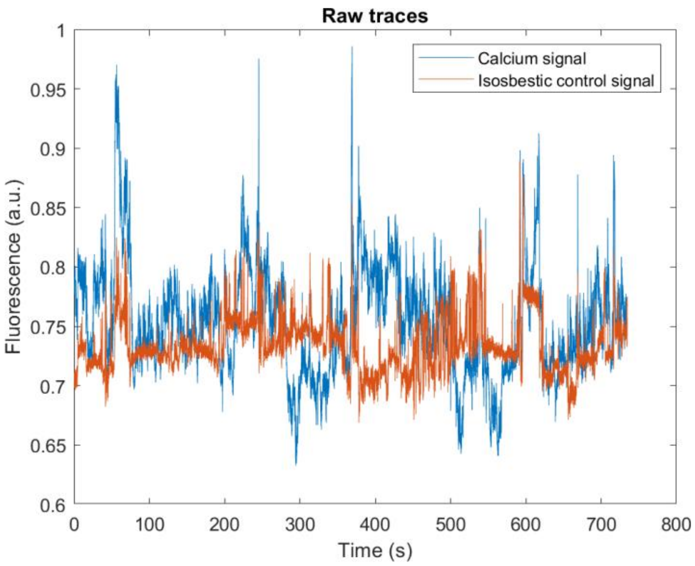

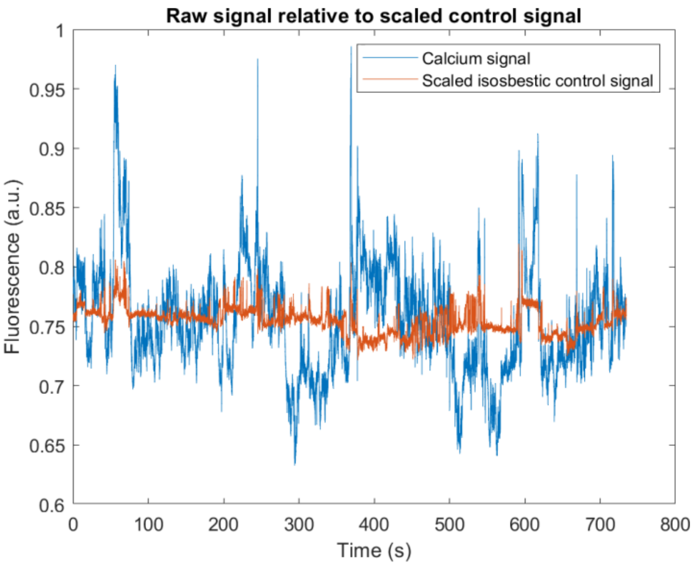

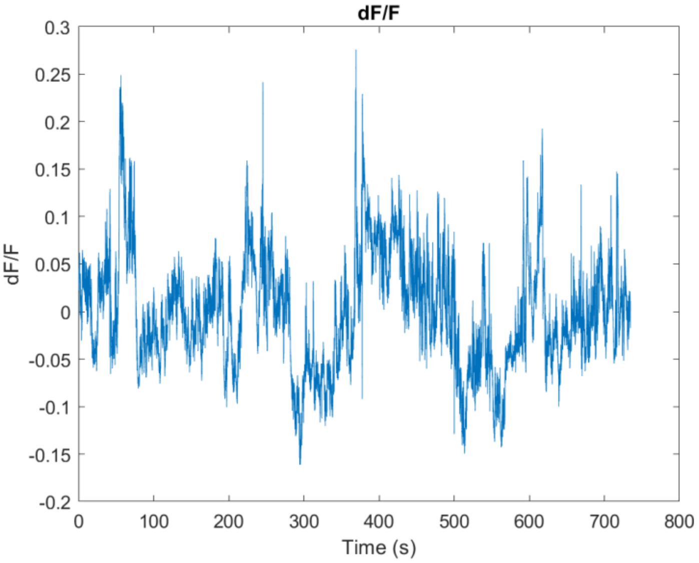

Same data as shown in Figures 1D,G

Mouse #611926

Session ID: 210529_Rec2

Lighting: Light

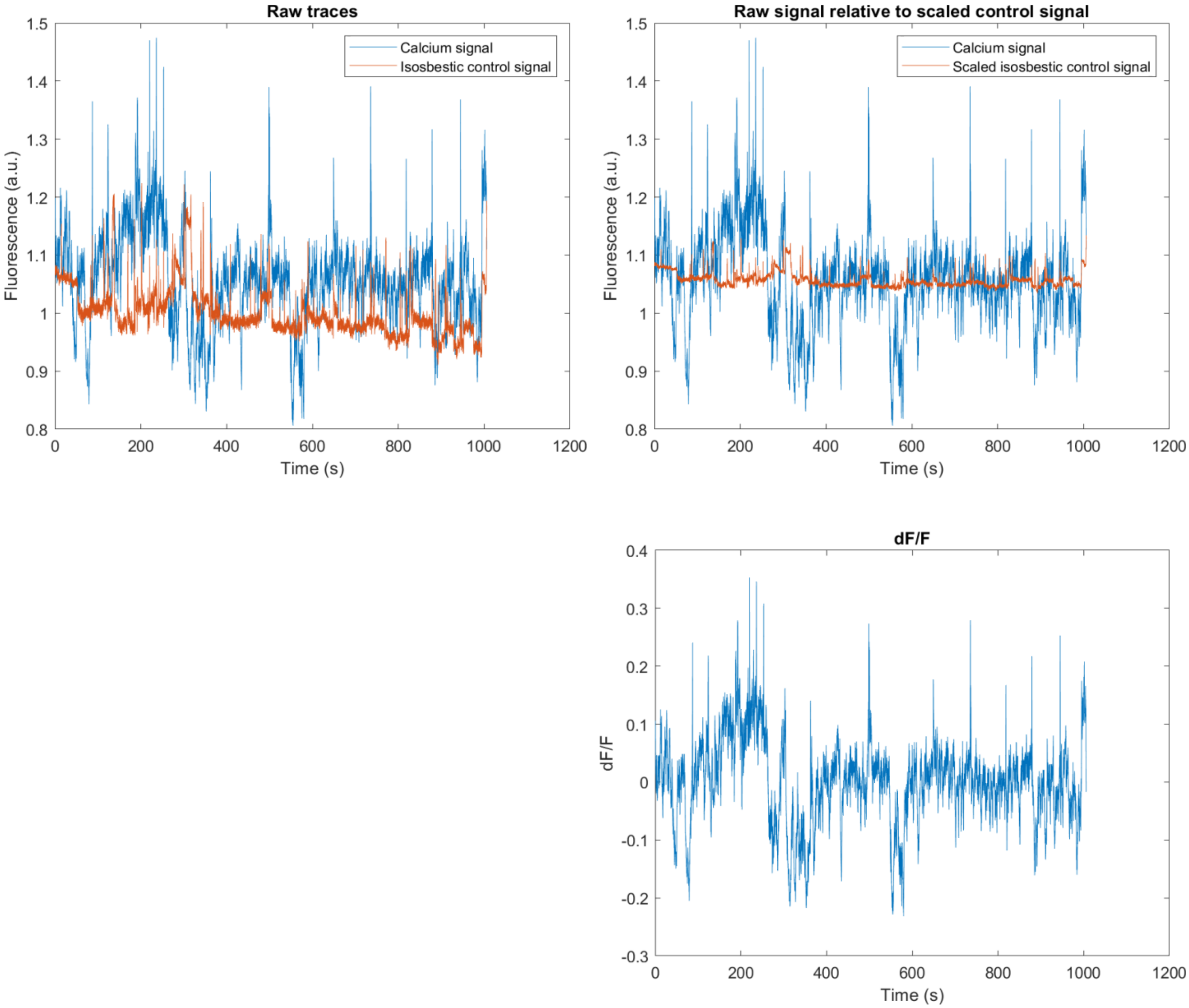

Mouse #611926
Session ID: 210528_Rec2
Lighting: Light

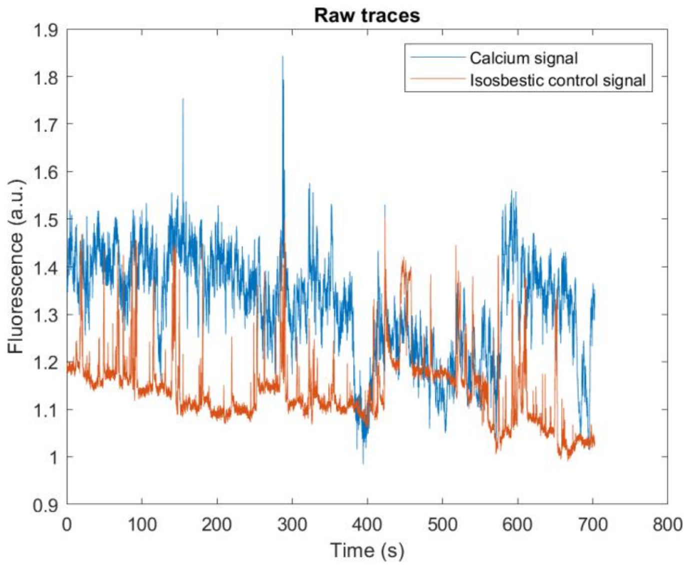

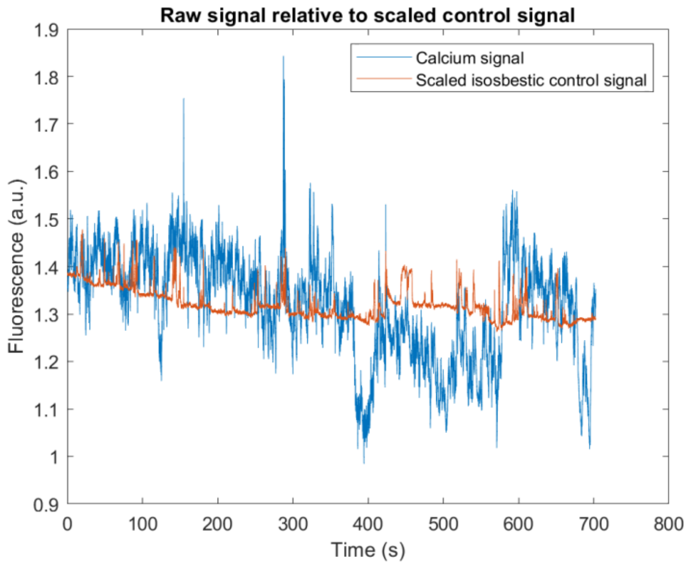

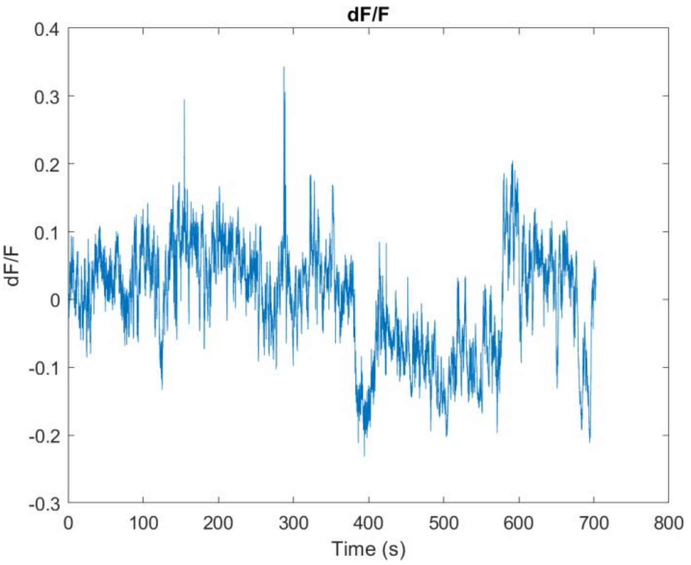

Mouse #616195
Session ID: 210205_Rec2
Lighting: Dark

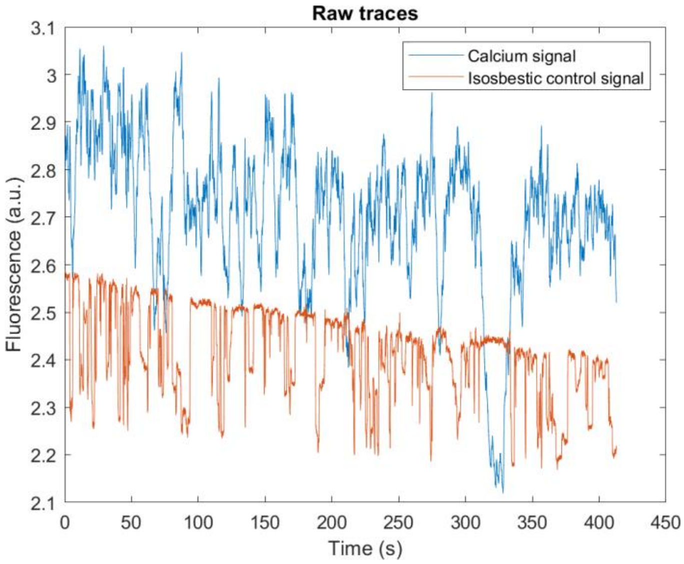

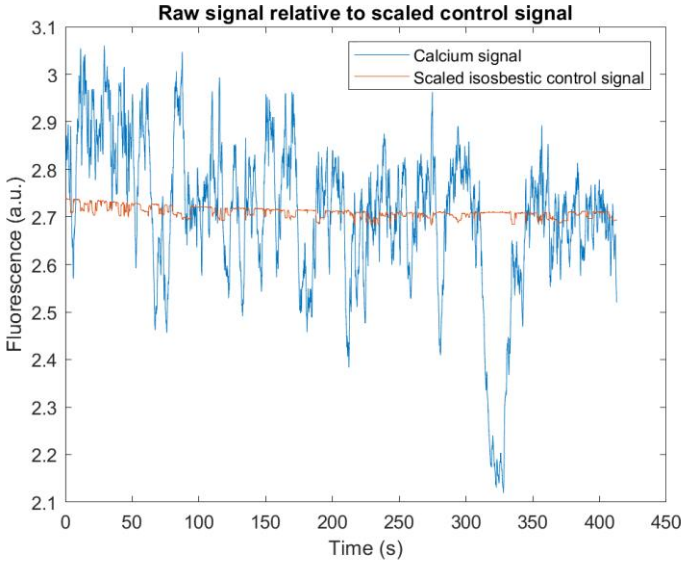

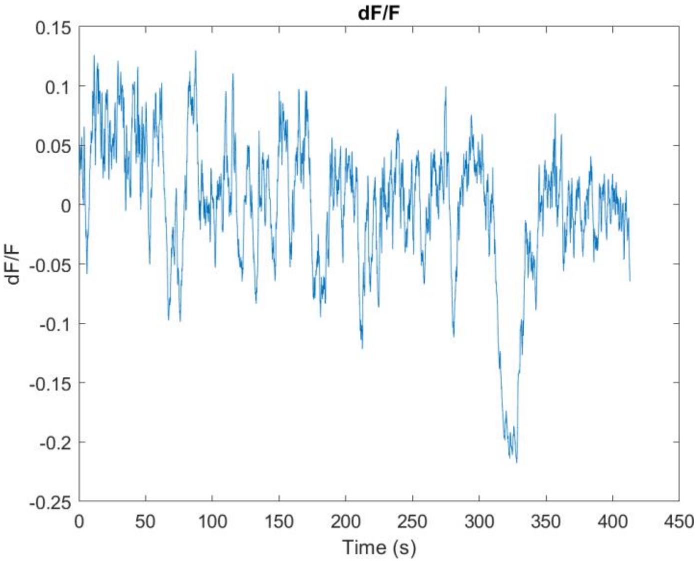

Same data as shown in Figures 2A,D

Mouse #616195

Session ID: 210129_Rec2

Lighting: Light

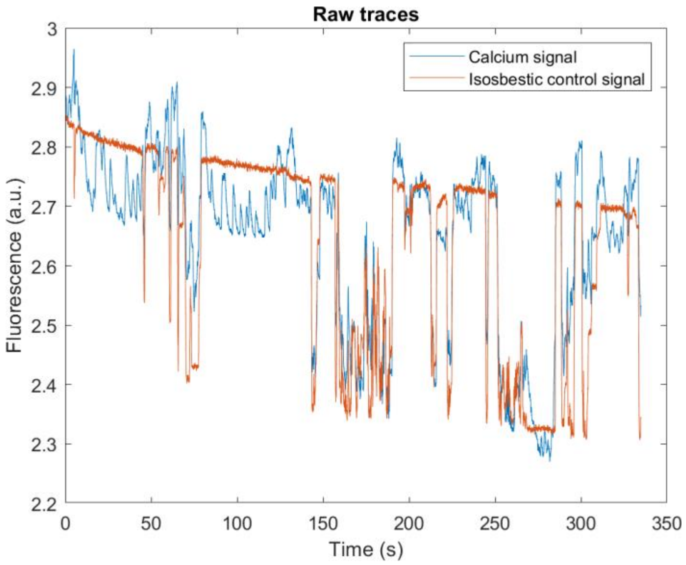

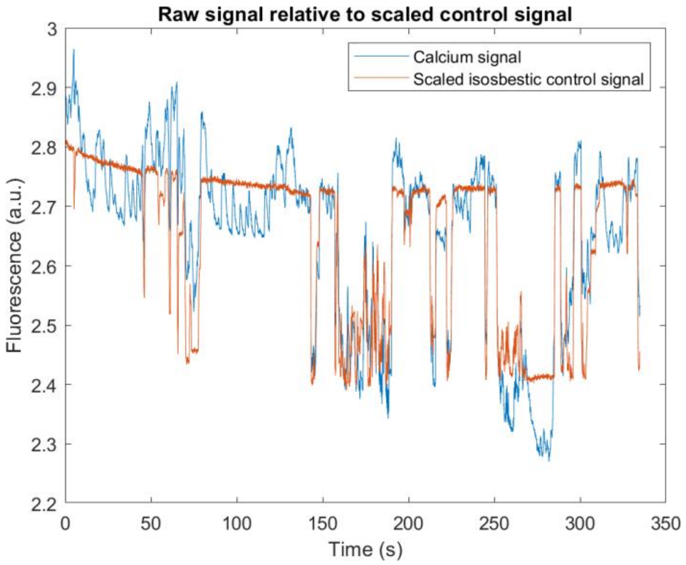

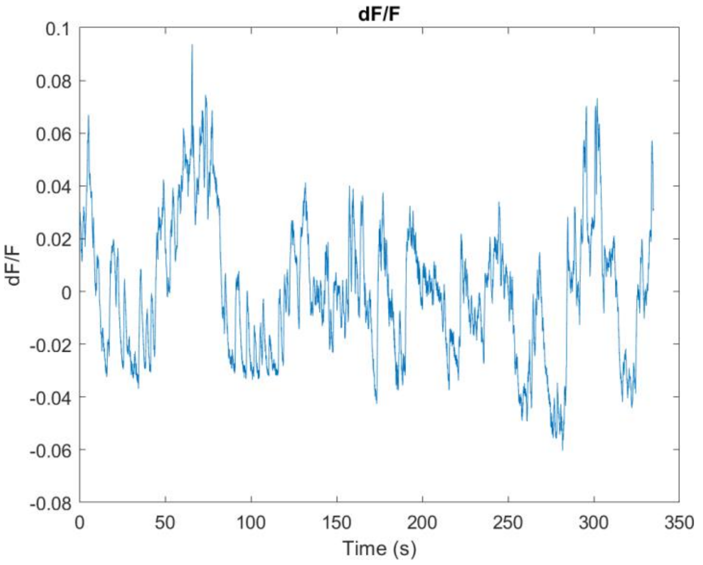

Mouse #616211
Session ID: 210326_Rec4
Lighting: Light

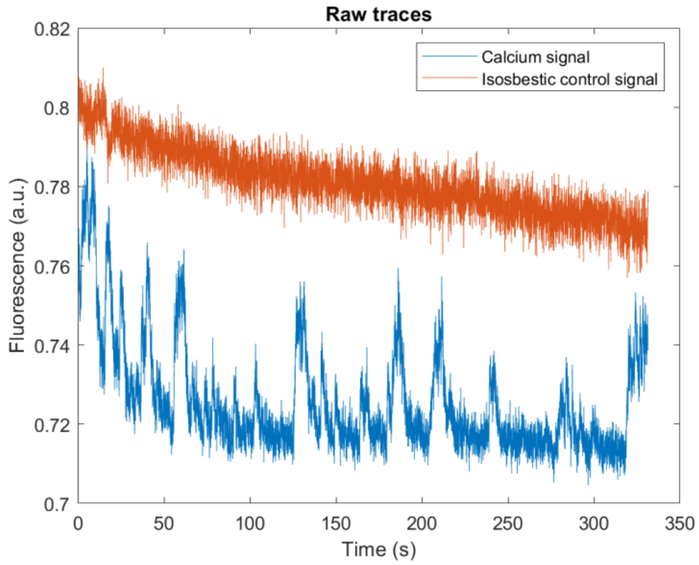

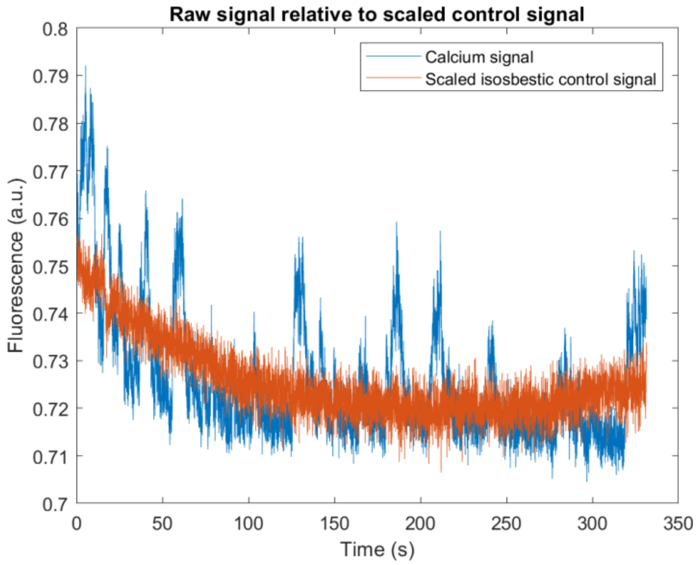

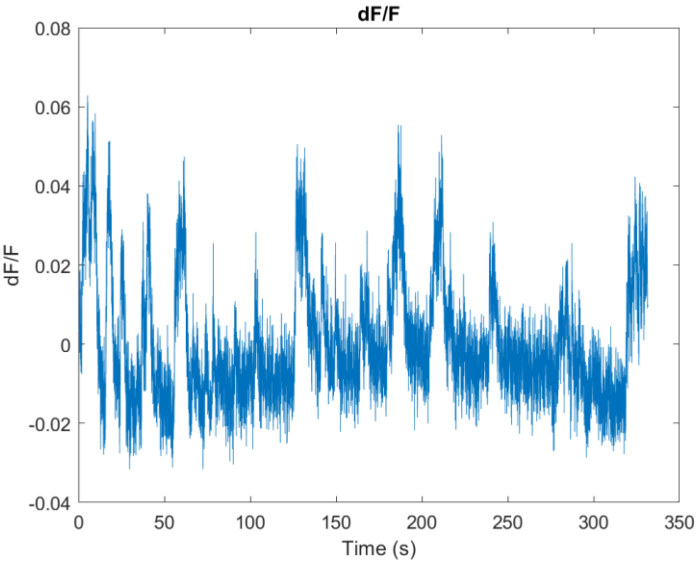

Mouse #616211
Session ID: 210324_Rec2
Lighting: Dark

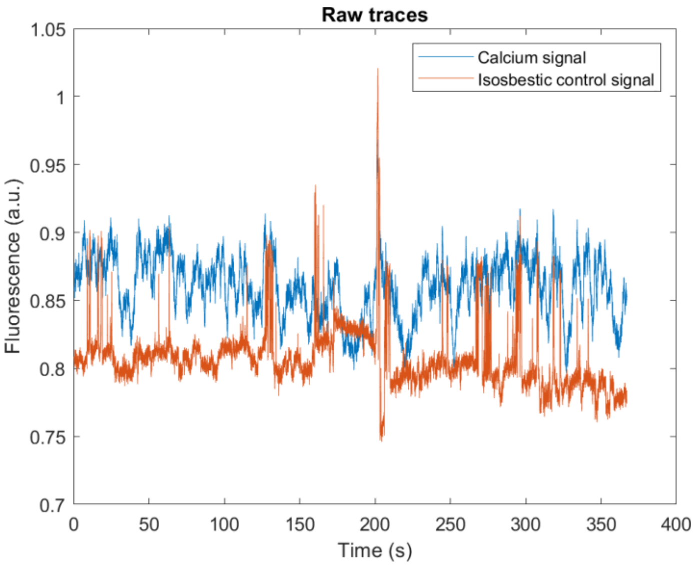

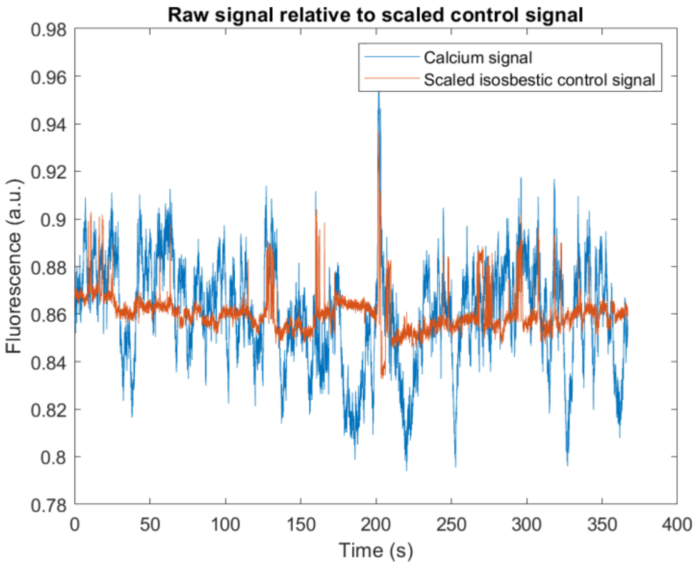

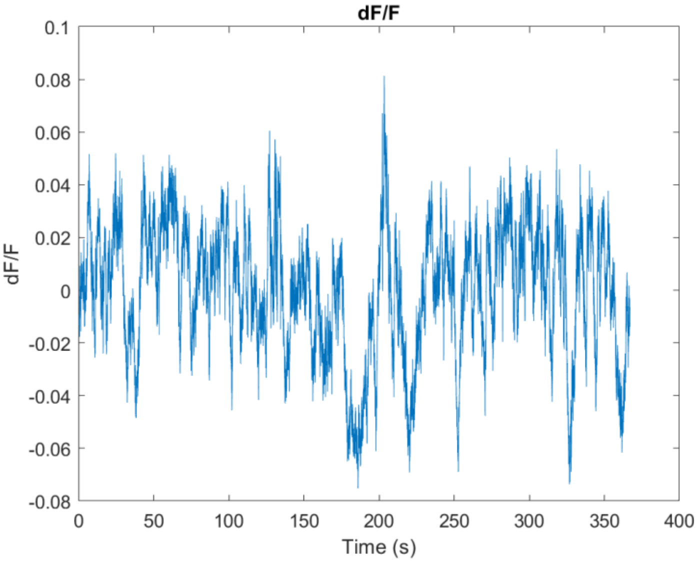

Supplement: Supplementary file 7 [file Data_Sheet_1.PDF]
